# MKS5 and CEP290 Dependent Assembly Pathway of the Ciliary Transition Zone

**DOI:** 10.1371/journal.pbio.1002416

**Published:** 2016-03-16

**Authors:** Chunmei Li, Victor L. Jensen, Kwangjin Park, Julie Kennedy, Francesc R. Garcia-Gonzalo, Marta Romani, Roberta De Mori, Ange-Line Bruel, Dominique Gaillard, Bérénice Doray, Estelle Lopez, Jean-Baptiste Rivière, Laurence Faivre, Christel Thauvin-Robinet, Jeremy F. Reiter, Oliver E. Blacque, Enza Maria Valente, Michel R. Leroux

**Affiliations:** 1 Department of Molecular Biology and Biochemistry and Centre for Cell Biology, Development and Disease, Simon Fraser University, Burnaby, British Columbia, Canada; 2 School of Biomolecular & Biomedical Science, University College Dublin, Belfield, Dublin 4, Ireland; 3 Department of Biochemistry and Biophysics, Cardiovascular Research Institute, University of California, San Francisco, San Francisco, California, United States of America; 4 Neurogenetics Unit, Mendel Laboratory, IRCCS Casa Sollievo della Sofferenza, San Giovanni Rotondo, Italy; 5 EA4271 GAD Génétique des Anomalies du Développement, FHU-TRANSLAD, Université Fédérale Bourgogne Franche-Comté, Dijon, France; 6 Service de Génétique clinique, CHU Reims, Reims, France; 7 Service de Génétique clinique, CHRU Strasbourg, Strasbourg, France; 8 Laboratoire de Génétique moléculaire, Plateau Technique de Biologie, CHU Dijon, Dijon, France; 9 Centre de Génétique, FHU-TRANSLAD, Hôpital d’Enfants, CHU Dijon, Dijon, France; 10 Department of Medicine and Surgery, University of Salerno, Salerno, Italy; Institut Curie, FRANCE

## Abstract

Cilia have a unique diffusion barrier (“gate”) within their proximal region, termed transition zone (TZ), that compartmentalises signalling proteins within the organelle. The TZ is known to harbour two functional modules/complexes (Meckel syndrome [MKS] and Nephronophthisis [NPHP]) defined by genetic interaction, interdependent protein localisation (hierarchy), and proteomic studies. However, the composition and molecular organisation of these modules and their links to human ciliary disease are not completely understood. Here, we reveal *Caenorhabditis elegans* CEP-290 (mammalian Cep290/Mks4/Nphp6 orthologue) as a central assembly factor that is specific for established MKS module components and depends on the coiled coil region of MKS-5 (Rpgrip1L/Rpgrip1) for TZ localisation. Consistent with a critical role in ciliary gate function, CEP-290 prevents inappropriate entry of membrane-associated proteins into cilia and keeps ARL-13 (Arl13b) from leaking out of cilia via the TZ. We identify a novel MKS module component, TMEM-218 (Tmem218), that requires CEP-290 and other MKS module components for TZ localisation and functions together with the NPHP module to facilitate ciliogenesis. We show that TZ localisation of TMEM-138 (Tmem138) and CDKL-1 (Cdkl1/Cdkl2/Cdkl3/Cdlk4 related), not previously linked to a specific TZ module, similarly depends on CEP-290; surprisingly, neither TMEM-138 or CDKL-1 exhibit interdependent localisation or genetic interactions with core MKS or NPHP module components, suggesting they are part of a distinct, CEP-290-associated module. Lastly, we show that families presenting with Oral-Facial-Digital syndrome type 6 (OFD6) have likely pathogenic mutations in CEP-290-dependent TZ proteins, namely Tmem17, Tmem138, and Tmem231. Notably, patient fibroblasts harbouring mutated Tmem17, a protein not yet ciliopathy-associated, display ciliogenesis defects. Together, our findings expand the repertoire of MKS module-associated proteins—including the previously uncharacterised mammalian Tmem80—and suggest an MKS-5 and CEP-290-dependent assembly pathway for building a functional TZ.

## Abstract 2

The transition zone is a barrier structure required to maintain the dynamic composition and functional integrity of the cilium. This study describes the pathway by which the transition zone is assembled during cilium formation.

## Author Summary

The primary cilium is a structure found in most animal cell types. Much like an antenna, it is responsible for sensing extracellular signals, including light and small molecules, and conveying this information to the receiving cell and respective tissue or organ. At the base of the cilium is the transition zone (TZ), which acts as a “gate” to regulate the entry and exit of ciliary proteins required for signal transduction. Here, we use the nematode *Caenorhabditis elegans* as a model system to dissect how different proteins within the TZ assemble to form a functional barrier. We find that the TZ protein MKS-5 (Rpgrip1/Rpgrip1L orthologue) forms the foundation for two different assembly pathways involving two distinct modules: Nephronophthisis (NPHP) and Meckel syndrome (MKS). We show that at the base of the MKS module is CEP-290, another TZ protein that assembles MKS module proteins, including a novel TZ protein we identify as TMEM-218. CEP-290 also helps assemble a potentially separate submodule containing TMEM-138 and CDKL-1. Notably, we provide evidence that the MKS module protein TMEM-17 facilitates cilium formation and is disrupted in the human disorder (ciliopathy) Oral-Facial-Digital Syndrome type 6 (OFD6). Together, our findings provide essential insights into the assembly pathway of the ciliary TZ and suggest further connections between the transition zone and human health.

## Introduction

The eukaryotic cilium represents a functionally diverse organelle whose microtubule-based axoneme is templated from a modified centriole-termed basal body [1]. Motile cilia (also called flagella) propel cells or generate flow across cell surfaces, and their dysfunction results in primary ciliary dyskinesia [2]. Nonmotile or primary cilia are present in most metazoan cell types and enable sensory processes, including chemosensation/olfaction, mechanosensation, and photosensation [3,4]. In vertebrates, primary cilia are associated with several signalling pathways (Hedgehog, Wnt, PDGF, cyclic nucleotide) [5–8]. Since primary cilia have pervasive roles in signalling, disruption of their function is linked to numerous human disorders (ciliopathies) that affect sensory physiology (vision, smell, hearing), as well as the development and function of most organs, including eyes, kidney, skeleton, and brain [4,9–12].

Several ciliopathies, such as Jeune asphyxiating thoracic dystrophy (JATD) and Bardet-Biedl syndrome (BBS), result from defects in the evolutionarily conserved intraflagellar transport (IFT) machinery [13–16]. IFT is responsible for the formation and maintenance of cilia. It uses kinesin anterograde motor(s) to mobilise cargo (e.g., structural components, receptors) from the basal body transition fibres to the ciliary tip, and a dynein retrograde motor to recycle components back to the base [17–19]. The IFT machinery comprises over 20 core components and a cargo-adaptor protein complex (BBSome) consisting of several BBS proteins [13,18]. Defects in core IFT and BBS proteins cause several multisystemic ailments, including cystic kidney disease, retinal degeneration, obesity, and skeletal malformations [20].

Another large macromolecular complex linked to a growing number of ciliopathies is the transition zone (TZ). The TZ represents the proximal-most domain of the ciliary axoneme, found immediately distal to the basal body [21–24]. Its ultrastructure, as observed by transmission electron microscopy (TEM), reveals doublet microtubules that are connected to the ciliary membrane, typically via Y-shaped structures [25]. These Y-links likely organise the so-called ciliary necklace, a repeating unit of membrane-associated proteinaceous “beads” that form rings or a spiral at the TZ membrane surface [21–23]. As detailed below, the Y-links and, presumably, the associated necklace appear to perform two principal functions: one in cilium formation, and the other as a ciliary “gate” that maintains the correct composition of the ciliary organelle.

TZ-associated ciliopathies include Meckel syndrome (MKS), Nephronophthisis (NPHP), Joubert syndrome (JBTS), Leber Congenital Amaurosis (LCA), and Senior Løken syndrome (SLNS) [10,26]. Collectively, the clinical ailments of these ciliopathies partially overlap with those arising from IFT/BBS dysfunction and include brain malformations, cystic kidneys, retinal dystrophy, liver fibrosis, and polydactyly. Many genes encoding TZ-localised proteins are mutated in one or more ciliopathies; however, for most ciliopathies, not all causative genes have been identified, suggesting that additional TZ proteins remain to be discovered [26,27].

Genetic interaction studies of TZ genes in *Caenorhabditis elegans* have defined two major functional modules, termed “MKS module” and “NPHP module.” The MKS module includes MKS-1 (mammalian Mks1 orthologue), MKSR-1 (B9d1/Mksr1), MKSR-2 (B9d2/Mksr2), MKS-2 (Mks2/Tmem216), MKS-3 (Mks3/Tmem67/Meckelin), MKS-6 (Mks6/Cc2d2a), TMEM-231 (Tmem231), JBTS-14 (Jbts14/Tmem237), TMEM-107, and TCTN-1 [25,28–32]. The *C*. *elegans* NPHP module thus far includes NPHP-1 and NPHP-4 [33]. Genetic (synthetic) interactions between the two modules are apparent. If one or more gene(s) within one module (either MKS or NPHP) are disrupted, ciliogenesis is essentially normal, although a subset of cilia are modestly truncated in *nphp-4* mutants [29,34]; in contrast, deleting individual genes from both modules severely disrupts TZ/ciliary structures [28–30,35,36]. For example, the *mks-6;nphp-4* double mutant has prominent ciliary phenotypes not observed in the individual single mutants [25,35]. These phenotypes include significantly fewer or no observable TZ Y-links, loss of basal body-transition fibre membrane attachments, and axonemal defects (missing or shorter microtubules) [25,28,30]. Hence, MKS and NPHP module proteins play genetically redundant roles in anchoring the basal body-ciliary axoneme and forming an ultrastructurally normal, functional TZ. Another established TZ protein, MKS-5 (RPGRIP1L/MKS5), is thought to act as a “scaffold” or “assembly factor” for most, if not all, MKS and NPHP module proteins [25,28,30,31].

Importantly, the genetically defined MKS and NPHP modules are consistent with physical interaction networks obtained through proteomic (pull-down) studies in mammalian cells, in which complexes containing the same (orthologous) MKS module proteins, or NPHP proteins, are observed [22,37–41]. In both *C*. *elegans* and mammalian cells, the modules can be further confirmed and defined hierarchically by testing for interdependent protein localisation at the TZ. Interestingly, mammalian Mks5 is found in a complex with Nphp1/Nphp4 proteins [39], a functional association not yet observed in *C*. *elegans*. Also, in *C*. *elegans* and mammalian cells, the TZ is thought to form early during ciliogenesis, following the docking of the basal body to either a ciliary vesicle or the plasma membrane, where the incipient ciliary axoneme is subsequently extended in an IFT-dependent manner [13,21,25,42].

Early evidence that the TZ forms a membrane diffusion barrier which concentrates signalling machinery within cilia came from Musgrave and colleagues (1986) [43], who presented cytological evidence suggesting that the unique composition of *Chlamydomonas eugametos* motile cilia is maintained by a membrane diffusion barrier. Spencer and colleagues (1988) [44] obtained similar evidence, showing that sequestration of rhodopsin to the outer segment of ciliary photoreceptors requires the TZ (connecting cilium). Together, these studies hinted at a “gate” or “barrier,” but until recently, the components involved and the mechanistic basis of this TZ functionality remained unknown.

At least 14 evolutionarily conserved TZ-localised proteins are now implicated in the formation of a selective membrane diffusion barrier that precludes nonciliary proteins from entering and helps to compartmentalise ciliary proteins [21,22,45]. In *C*. *elegans*, two membrane-associated proteins, RPI-2 (orthologue of Retinitis Pigmentosa 2) and TRAM-1 (Translocating Chain-Associating Membrane Protein) are present at the base of cilia and are excluded from the ciliary compartment unless TZ protein(s) are disrupted [25]. In mammals, several signalling proteins (e.g., Adcy3, Arl13b, Inpp5e, Sstr3, Pkd2, and Smo) either fail to enter cilia or are no longer concentrated within the organelle in various individual TZ mutants [31,38,40]. Similarly, the ciliary composition of cilia is altered in the TZ mutants of *Chlamydomonas* (*cep290* and *nphp4*), *Drosophila* (*Cep290*), and ciliary photoreceptor (*Rpgr*) [46–49].

Although many TZ components have been identified and their role in establishing a ciliary gate is generally accepted, many questions regarding the TZ remain unanswered. Are there additional components of the TZ awaiting discovery, and do they fit within the known MKS or NPHP functional modules? Aside from Mks5/Rpgrip1L, are there other essential “core” scaffolding and/or assembly factors of the MKS and/or NPHP modules at the TZ? Finally, how are the different proteins and modules spatiotemporally assembled at the TZ?

In this study, we provide new insights into the composition, organisation, assembly, and function of the *C*. *elegans* TZ, as well as provide evidence for an expanded role for TZ proteins in ciliopathies. We identify *C*. *elegans* TMEM-218 (mammalian Tmem218) as a novel TZ protein. Our genetic interaction, hierarchical, and in vivo functional analyses reveal that TMEM-218 is a new MKS module component. We show that, in addition to *C*. *elegans* MKS-5 (Rpgrip1L/Rpgrip1), another TZ protein, CEP-290 (Cep290/Mks4/Nphp6), functions as an assembly factor for not only TMEM-218 but all MKS module proteins tested. Consistent with a key role for CEP-290 in establishing TZ ultrastructure, the *cep-290* mutant lacks all characteristic features of a TZ, including Y-links. Notably, removal of CEP-290 does not significantly perturb the localisation of NPHP module proteins or MKS-5, whereas removal of MKS-5 results in CEP-290 delocalisation. Our findings suggest an assembly pathway that is initiated by MKS-5 and involves two separate branches. One branch requires CEP-290 for the assembly of MKS module proteins as well as two other TZ proteins (TMEM-138 and Cyclin-Dependent Kinase-Like CDKL-1) that may form a separate module. The other branch of the pathway, which can assemble separately, involves the NPHP module. Finally, we present evidence that three human genes encoding CEP-290-dependent TZ components, *TMEM17*, *TMEM138*, and *TMEM231*, are mutated in Oral-Facial-Digital type 6 (OFD6) syndrome families; furthermore, a novel mammalian TZ protein we uncovered, Tmem80, represents an excellent ciliopathy candidate. Notably, *TMEM17* has not yet been associated with a human disorder, and patient-derived cells with the *TMEM17* mutation display impaired ciliogenesis. Collectively, our work provides essential insights into the formation, organisation, and function of the TZ and expands the number of likely or potential ciliopathy-associated TZ proteins.

## Results

### *C*. *elegans tmem-218* Encodes a Novel Ciliary Transition Zone Protein and Is Genetically Associated with the MKS Module

**Fig 1:**
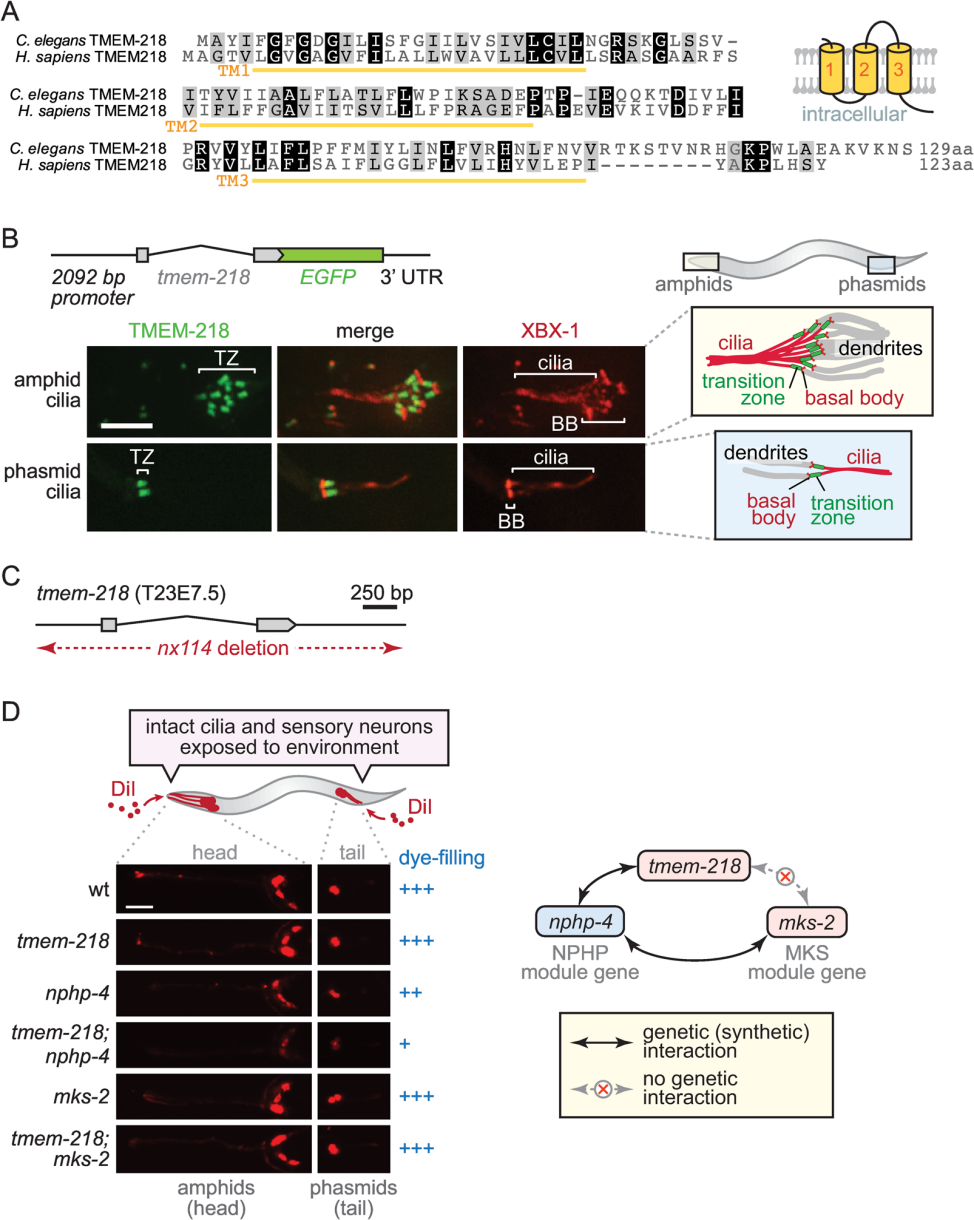
*C*. *elegans tmem-218* encodes a novel transition zone protein and genetically interacts with *nphp-4* but not *mks-2*, consistent with it being part of the Meckel syndrome (MKS) module. (**A**) Amino acid sequence alignment of *C*. *elegans* TMEM-218 and its *Homo sapiens* orthologue. Both proteins are predicted to encode proteins with three transmembrane domains (TM1-3), with the predicted topology shown schematically. (**B**) *C*. *elegans tmem-218* (T23E7.5) is expressed specifically in ciliated sensory neurons and its encoded product (TMEM-218) tagged with green fluorescent protein (GFP; green) localises to the ciliary transition zone (TZ) at amphid (head) and phasmid (tail) cilia found at the distal ends of dendrites. The tdTomato-tagged intraflagellar transport (IFT) protein XBX-1 (red) marks basal bodies (BB) and ciliary axonemes. Scale bar, 4 μm. (**C**) The *tmem-218(nx114)* mutant allele used in this study, generated using transposon-mediated mutagenesis, represents a complete and specific deletion of the *tmem-218* gene. (**D**) Dye-filling assay schematic shows the fluorescent dye DiI (red) penetrating sensory neurons via structurally intact, environmentally exposed ciliated endings. Dye-filling of the *tmem-218* and *nphp-4* strains is normal (+++) or slightly reduced (++), respectively, whereas dye-filling of the *tmem-218;nphp-4* double mutant is mostly disrupted (+). A lack of genetic (synthetic) interaction between *tmem-218* and *mks-2* is also consistent with TMEM-218 belonging to the MKS module (as depicted schematically). Scale bar, 40 μm.

Recently, a large-scale mutagenesis screen uncovered a knockout mouse strain with NPHP and retinal degeneration phenotypes reminiscent of SLSN (OMIM 266900), a known ciliopathy [50]. Although this strain was found to harbour a mutation in Tmem218, the protein was not characterised at the molecular or cellular level. Tmem218 encodes a small protein with three transmembrane domains that is conserved across metazoans, including *C*. *elegans* and humans (Fig 1A). Tmem218 is detectable in at least some, if not all, ciliated protists, including the green algae *Chlamydomonas reinhardtii*, the Chromalveolate *Guillardia theta*, and Choanoflagellates, the most closely related unicellular metazoan ancestors.

To determine if the unstudied *C*. *elegans* orthologue of Tmem218 (TMEM-218) plays a cilium-associated role, we generated an expression construct consisting of the endogenous promoter and entire coding region fused in-frame to green fluorescent protein (GFP). Transgenic strains expressing this construct were observed by confocal microscopy to ascertain which cell types the gene is expressed in. We found that *tmem-218* is expressed exclusively in cells that possess cilia, including amphid (head) and phasmid (tail) sensory neurons (Fig 1B). This expression pattern is like that of most *C*. *elegans* ciliary genes, including those encoding IFT, BBS, and TZ proteins [3,15,25,36,51]. The GFP-tagged TMEM-218 protein is specifically concentrated at the base of cilia, immediately distal to the basal body-associated transition fibres (Fig 1B; an IFT-dynein comarker, XBX-1::tdTomato, marks transition fibres and axoneme). This subcellular localisation for TMEM-218 is identical to known TZ proteins [25,28,31,35].

Next, we investigated whether TMEM-218 could be assigned to one of the two established genetic modules, MKS or NPHP [25]. Since a null mutant allele of *tmem-218* was not available from knockout consortia or the Million Mutation Project (MMP), we used transposon-mediated mutagenesis (imprecise excision) to generate one. Using this approach, we uncovered a deletion allele, *tmem-218(nx114)*, that removes the entire coding region of *tmem-218* without affecting neighbouring genes (Fig 1C). Mutant animals outcrossed to wild-type are viable and appear grossly normal in terms of morphology, movement, and development.

To determine if *tmem-218* displays genetic interactions with other TZ genes, we subjected single and double mutant strains to a dye-filling assay that tests for cilia structure defects [51–53]. Wild-type animals incubated with fluorescent DiI solution display dye-filling in several head (amphid) and tail (phasmid) sensory neurons, indicating normal exposure of cilia to the external environment (see schematic, Fig 1D). As with all previously tested TZ mutants, the *tmem-218* single mutant shows normal dye-filling (Fig 1D). However, a double mutant with *tmem-218* and an NPHP module mutant, *nphp-4*, shows a prominent dye-filling phenotype (Fig 1D). Such a genetic interaction is consistent with TMEM-218 acting together with NPHP-4 to facilitate TZ and cilium ultrastructure formation, a possibility that will need to be confirmed by TEM analysis. We also show that, as expected for an MKS module mutant [25], combining the *tmem-218* mutation with another MKS module mutant, *mks-2* [28], does not cause a dye-filling phenotype (Fig 1D). Altogether, our findings that *tmem-218* encodes a TZ protein and genetically interacts with *nphp-4* but not *mks-2* are consistent with TMEM-218 representing a novel MKS module protein.

### TMEM-218 Localisation at the Transition Zone Depends on MKS-5 and MKS Module Proteins

**Fig 2:**
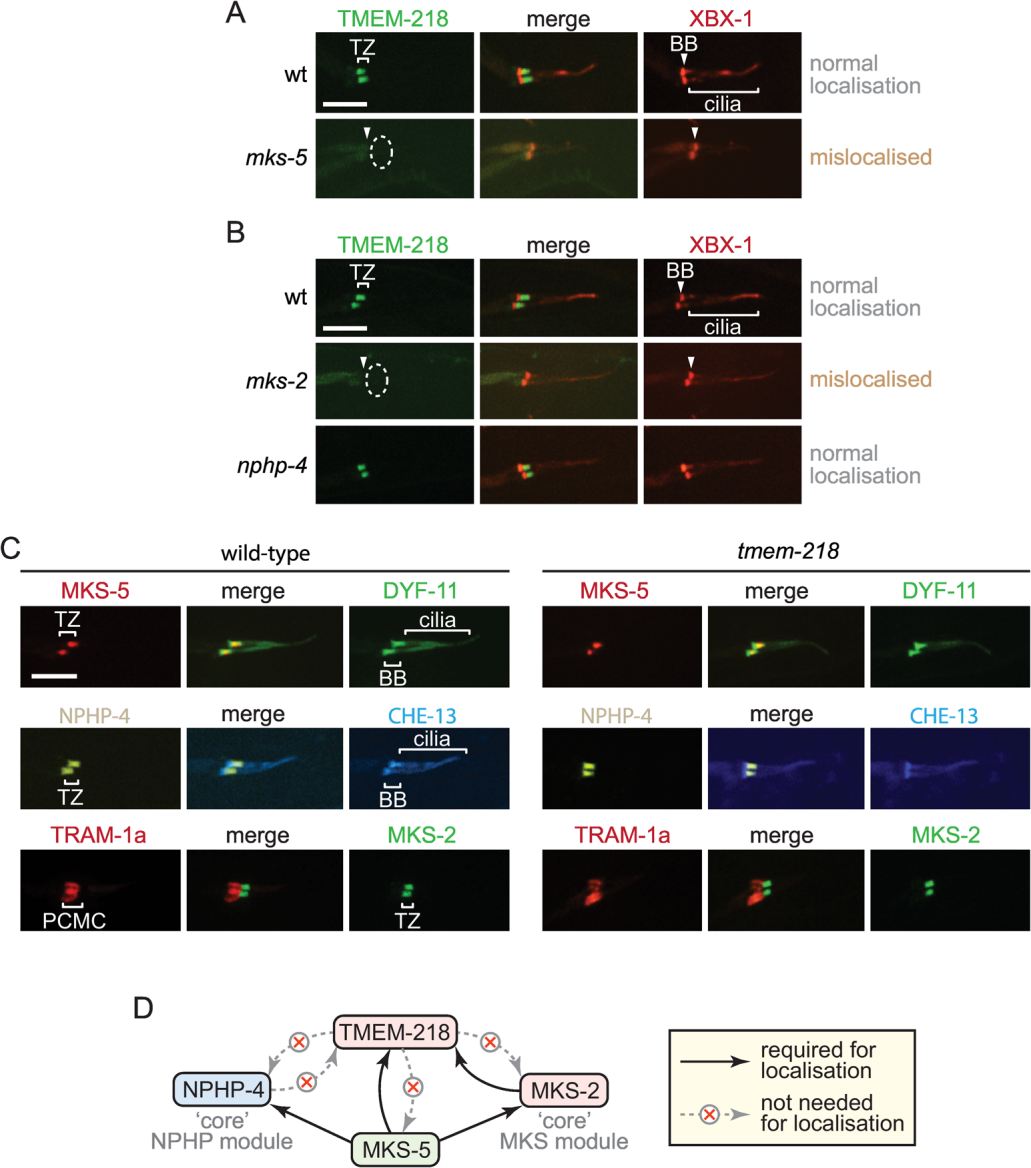
TMEM-218 depends on MKS-5 and a core MKS module component (MKS-2) for transition zone localisation but does not itself influence other TZ proteins. (**A**) GFP-tagged TMEM-218 (green) localises to the transition zone (TZ) normally in a wild-type background and is mislocalised in the *mks-5* mutant (dotted ellipse). The tdTomato-tagged IFT protein XBX-1 (red) marks basal bodies (BB) and ciliary axonemes. Scale bars in panels **A–C** are 4 μm. (**B**) GFP-tagged TMEM-218 displays normal localisation in the core NPHP module mutant *nphp-4* but is mislocalised in the core MKS module mutant *mks-2* (dotted ellipse), consistent with TMEM-218 being part of the MKS module. (**C**) In the *tmem-218* mutant, MKS-5 (tdTomato-tagged; red), the MKS module protein MKS-2::GFP (green), and the NPHP module protein NPHP-4::YFP (yellow) are correctly localised to the TZ. DYF-11::GFP (green), CHE-13::CFP (blue), and XBX-1::tdTomato (red) are IFT proteins that mark the basal body (BB) and ciliary axoneme. TRAM-1a::tdTomato (red) is a periciliary membrane compartment (PCMC) protein that localises correctly in the *tmem-218* mutant, suggesting that TMEM-218 is not necessary for TZ gate function. (**D**) Localisation-dependence studies are consistent with TMEM-218 being a peripherally-associated MKS module protein of the TZ.

Our previous studies on *C*. *elegans* TZ proteins revealed that MKS-5 (mammalian Rpgrip1L/Rpgrip1) plays a central role in assembling MKS module components at the TZ [25,28,30,31]. We therefore queried if MKS-5 is also required for the TZ localisation of TMEM-218. To test this, we introduced the TMEM-218::GFP fusion protein into the *mks-5* mutant. While TMEM-218::GFP is concentrated at the TZ in wild-type animals, it is consistently mislocalised (absent from the TZ) in the *mks-5* mutant background (Fig 2A). This finding supports the notion that TMEM-218 is functionally associated with the established network of TZ proteins, and confirms MKS-5 as a critical assembly factor for all known MKS module proteins tested thus far.

To provide additional evidence that TMEM-218 is specifically associated with the MKS module, we assessed whether its TZ localisation is perturbed upon disruption of a“core” MKS module protein. Indeed, TMEM-218 is no longer present at the TZ in the *mks-2* MKS module mutant (Fig 2B). In contrast, and as expected for an MKS module protein, TMEM-218 remains correctly localised in the *nphp-4* core NPHP module mutant (Fig 2B). Reciprocal experiments were performed to query if TMEM-218 is itself required for the localisation of other TZ proteins. Our data show that TMEM-218 is not required for the localisation of MKS-5, MKS-2 (“core” MKS module protein), or NPHP-4 (“core” NPHP module protein) (Fig 2C).

Overall, our findings point to a specific association between TMEM-218 and MKS module components, potentially as a more “peripheral” TZ component compared to other “core” MKS module proteins, which include MKS-2, MKSR-1, MKSR-2, and TMEM-231 (Fig 2D) [25,28,30,31]. Consistent with this possibility, these “core” TZ proteins are all necessary for TZ gate function—namely, restricting the inappropriate entry of membrane-associated TRAM-1a into cilia—but TMEM-218, similar to the “peripheral” TZ protein MKS-3 [25], does not appear to influence this aspect of TZ function (Fig 2C).

### *C*. *elegans* CEP-290 Plays an Essential Role in Transition Zone Function and Assembly

Given our discovery of a novel MKS module protein dependent on MKS-5 for TZ localisation, we wondered if there are additional proteins that participate in anchoring/assembling MKS module proteins at the TZ. We hypothesised that Cep290, which is implicated in several ciliopathies (including MKS and JBTS) [54] and is suggested to be a structural component of the TZ in *Chlamydomonas* [48], might represent such a protein. We sought to analyse the *C*. *elegans* homologue of Cep290, encoded by the gene Y47G6A.17.

**Fig 3:**
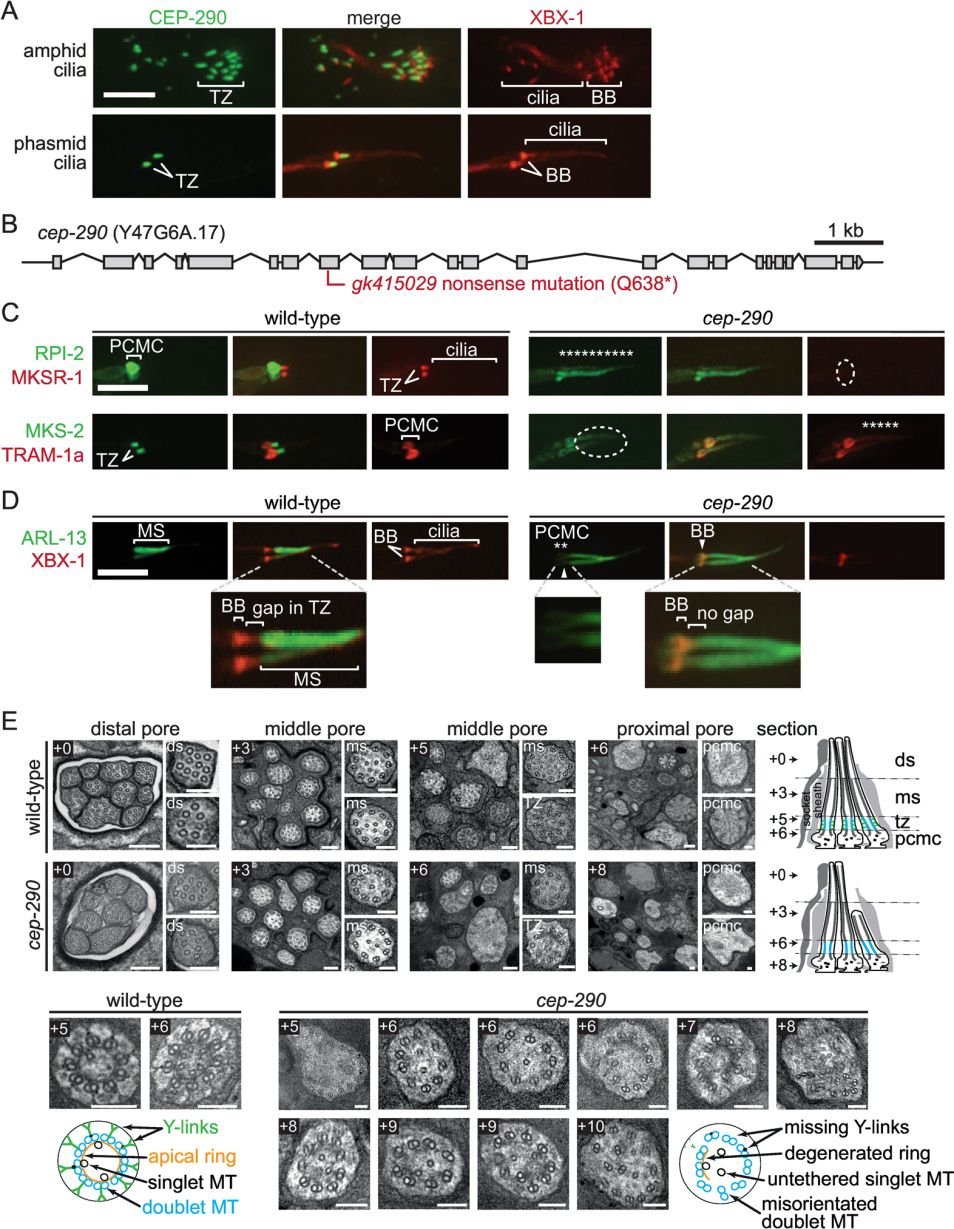
*C*. *elegans* CEP-290 is a transition zone protein required for ciliary gate (membrane diffusion barrier) function and transition zone assembly. (**A**) *C*. *elegans* CEP-290 (Y47G6A.17) tagged with GFP (green) is expressed only in ciliated sensory neurons (including amphids and phasmids) and is specifically enriched at the ciliary transition zone (TZ). tdTomato-tagged XBX-1 (red) is an IFT protein that marks the basal body (BB) and axoneme. Scale bars in panels **A, C,** and **D** are 4 μm. (**B**) *cep-290* gene structure and likely null allele (*gk415029* nonsense mutation) isolated from the Million Mutation Project (MMP) mutant strain VC30108. (**C**) CEP-290 is required for maintaining the functional integrity of the TZ. Two membrane-associated proteins (TRAM-1a::tdTomato and RPI-2::GFP) normally present at the base of cilia and not present within cilia show abnormal ciliary entry (“leakage”) or accumulation in the *cep-290* mutant (shown as asterisks). MKSR-1::tdTomato and MKS-2::GFP are TZ protein comarkers (in wild-type) that mislocalise in the *cep-290* mutant (dotted ellipse). (**D**) CEP-290 is required to exclude membrane-associated GFP-tagged ARL-13 (green) from the transition zone and compartmentalise it within the ciliary middle segment (MS), consistent with a role for CEP-290 in TZ gate function. In the *cep-290* mutant, ARL-13 is also seen outside of the cilium in the periciliary membrane compartment (PCMC) region (mislocalisation shown with asterisks). (**E**) Transition zone ultrastructure is missing in *cep-290* mutants. Transmission electron microscopy images from serial cross-sections of the amphid channel (pore) in wild-type and *cep-290* mutant worms. Boxed numbers denote positioning of sections relative to anterior-most section (+0); section positions also shown in schematics on right-hand side. Wild-type amphid channels possess ten ciliary axonemes, each with distal segment (ds; outer singlet A-tubules), middle segment (ms; outer doublet A/B tubules), and transition zone (TZ; outer doublet A/B tubules, Y-links, apical ring) compartments. In *cep-290* mutants, one to two cilia are missing from the distal pore, indicating that one to two axonemes are short. TZ ultrastructure is severely disrupted in *cep-290* worms; most or all Y-link structure is missing, as is the apical ring that draws together outer doublet MTs with varying numbers of inner singlet microtubules. MT doublets are also frequently disorganised near the ciliary base. Schematics denote the observed ultrastructural phenotypes (only three ciliary axonemes shown for simplicity). pcmc; periciliary membrane compartment. Scale bars, 200 nm (large, low-magnification top panel images) and 100 nm (small, high-magnification top panel images and all bottom panel TZ images).

To test our hypothesis, we created a construct encompassing the full-length cDNA of Y47G6A.17 fused in-frame to GFP. As anticipated, the Y47G6A.17::GFP translational fusion protein is specifically enriched at the TZ of all cilia (Fig 3A); importantly, we confirmed that the GFP-tagged protein is functional and, hence, its localisation is physiologically relevant (see below). Based on Y47G6A.17 consisting largely of coiled coils and having sequence homology to mammalian Cep290, its TZ localisation, and our finding that it plays an important role in the assembly and function of the TZ (see below), our data support the notion that it is indeed the functional homologue of Cep290; we therefore named the nematode protein CEP-290. Notably, Schouteden and colleagues also recently confirmed that *C*. *elegans* CEP-290 localises to the TZ [55].

To provide evidence that *C*. *elegans* CEP-290 is implicated in ciliary gate function, we first acquired a strain (VC30108) from the MMP [56] that contains a nonsense mutation (Q638*) in the coding region of *cep-290* (Y47G6A.17) (Fig 3B). Quantitative RT-PCR analyses show that the *cep-290* transcript in this mutant is eliminated by nonsense-mediated mRNA decay (NMD) (S1A Fig). The strain, *cep-290(gk415029)*, therefore very likely contains a null allele of *cep-290*, consistent with its ciliary phenotypes being indistinguishable from those described for a different strain harboring a complete loss-of-function allele of *cep-290* [55]; our strain was outcrossed to wild-type to remove unlinked background mutations.

We then tested for the abnormal entry of two membrane-associated proteins into the cilia of *cep-290* mutant animals. Both RPI-2 (mammalian RP2 orthologue) and TRAM-1a (TRAM in mammals) are normally excluded from cilia in wild-type animals (Fig 3C) [25]. In contrast, visualisation of fluorescently-tagged RPI-2 and TRAM-1a in the *cep-290* mutant consistently reveals their abnormal “leaking” or accumulation in cilia (Fig 3C). These findings parallel those found with several previous TZ mutants [25] and provide evidence that CEP-290 is required for the ciliary gating function of the TZ.

Further support for this possibility stems from our observation of ARL-13 (Arl13b in mammals), which freely diffuses within the ciliary membrane [45,57]. In wild-type animals, palmitoylated ARL-13 (and other membrane-associated proteins) are completely excluded from the TZ, a reflection of the TZ acting as a membrane diffusion barrier [30,45]. In the *cep-290* mutant, the ARL-13 ciliary zone of exclusion (CIZE) at the TZ is substantially disrupted, with ARL-13 visible within the proximal region of the cilium and at the periciliary membrane (Fig 3D); this is similar to that observed for ARL-13 and other membrane-associated proteins in the *mks-5* mutant or TZ double mutants [30,45]. Together, our two complementary TZ functional assays suggest that CEP-290 plays an essential role in maintaining a functional TZ “gate” or membrane diffusion barrier.

To assess the potential role of CEP-290 in assembling TZ ultrastructure, which would help explain its role in TZ gate function, we subjected the *cep-290* mutant to TEM analyses. In wild-type animals, the following structures are observed in TEM cross-sections of the amphid channel ciliary bundle: transition fibres that connect the distal end of the basal body to the base of the ciliary membrane, a TZ with prominent Y-links, a middle segment axoneme with doublet microtubules, and, finally, a distal segment with singlet microtubules (Fig 3E). In the *cep-290* mutant, most cilia are present throughout the pore, although one to two axonemes are missing from the distal regions, indicating that they are short (Fig 3E). Like wild-type worms, *cep-290* mutants also possess clearly distinguishable middle and distal segments. Strikingly, however, *cep-290* mutant cilia reveal no structures characteristic of the TZ, displaying a lack of Y-link axoneme-to-membrane attachments; furthermore, the apical ring structure (of unknown composition) that is present inside the doublet microtubules is also absent (Fig 3E; see also [55]). In addition, microtubule doublets are frequently disorganised near the ciliary base and in the periciliary membrane compartment of some neurons (Fig 3E). Notably, the *cep-290* null mutant TZ phenotype is essentially indistinguishable from that of the *mks-5* null mutant [30]. Together, our findings reveal a critical role for *C*. *elegans* CEP-290 in the formation of TZ ultrastructure as well as in ciliary gate function.

### *C*. *elegans* CEP-290 Is Essential for Assembly of TMEM-218 and MKS Module Components at the Transition Zone, Requires MKS-5 Coiled Coil Region for its Localisation, and Behaves Genetically as an MKS Module Component

**Fig 4:**
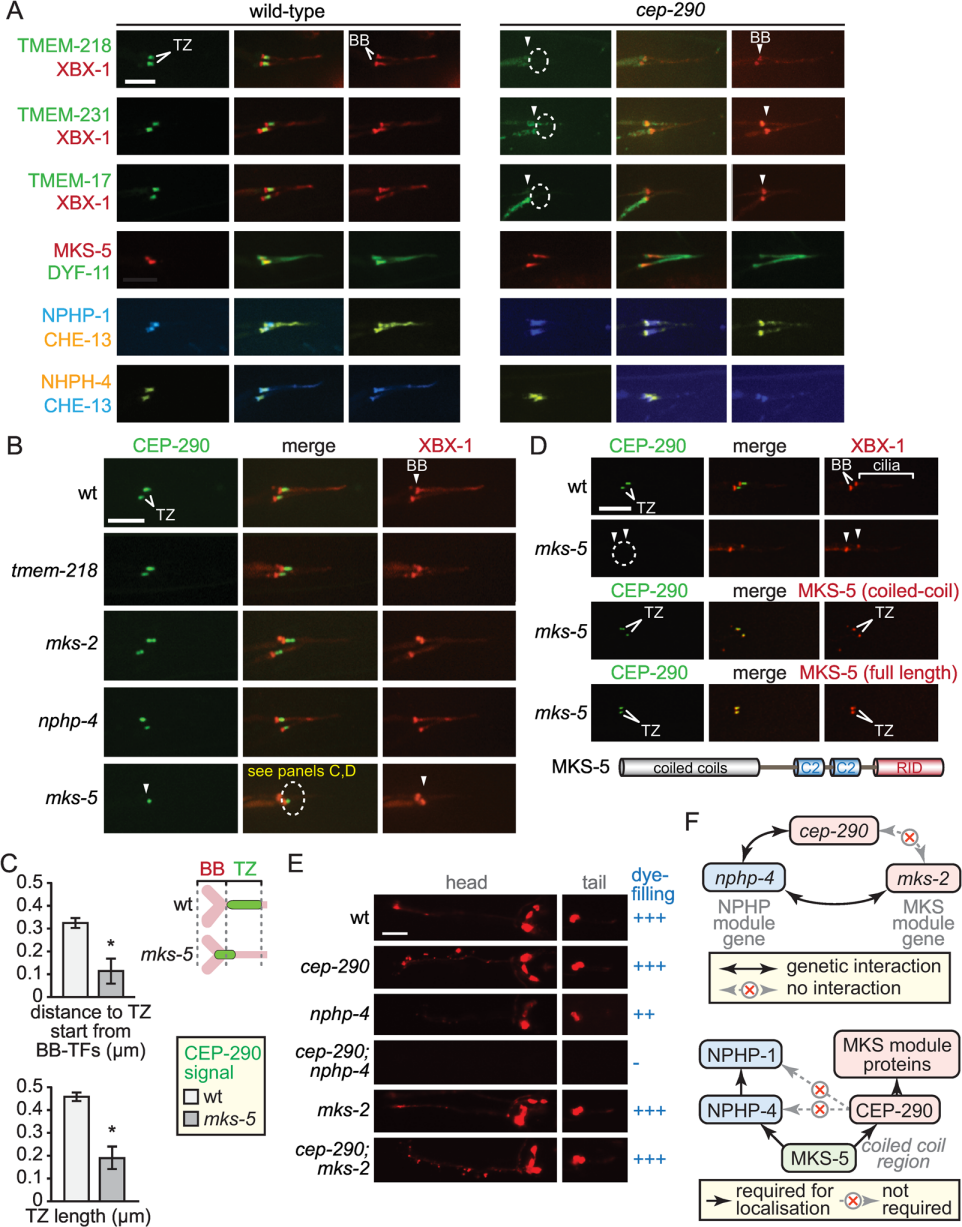
CEP-290 is specifically required for the assembly of the MKS module but not NPHP module components, and depends on MKS-5 for its own TZ localisation. (**A**) TMEM-218::GFP and other MKS module components (TMEM-231::GFP, TMEM-17::GFP) no longer localise to the TZ in the *cep-290* mutant (dotted ellipses). However, MKS-5::tdTomato and NPHP module proteins (NPHP-1::CFP, NPHP-4::YFP) retain their localisation at the base of cilia (in the same region as the TZ) in the *cep-290* mutant. Scale bars (panels **A and B**), 4 μm. (**B**) Transition zone localisation of GFP-tagged CEP-290 (green) requires MKS-5 (dotted ellipse) but is not affected by loss of MKS or NPHP module components. XBX-1::tdTomato (red) marks the basal body-transition fibres and axoneme. CEP-290 levels are reduced in the *mks-5* mutant, and remaining signals are closer to the basal body-transition fibres (see panel **C;** panel D shows complete loss of CEP-290 in the *mks-5* mutant). (**C**) Analysis of CEP-290 localisation in wild-type and *mks-5* mutant. Quantitation of CEP-290::GFP signal in the *mks-5* mutant reveals closer localisation to the basal body-transition fibre region (marked by XBX-1::tdTomato) and reduced signal length compared to that of wild-type animals. *, Fisher’s exact test for significance, *p* < 0.05. Schematic depicts differences in wild-type and *mks-5* mutant CEP-290::GFP localisation patterns. BB, basal body; TF, transition fibres. **(D)** Loss of CEP-290 TZ localisation in the *mks-5* mutant (dotted ellipse) is restored through expression of full length MKS-5, as well as the coiled coil domain of MKS-5 alone (refer to schematic for MKS-5 domain structure; RID, RPGR-interacting domain). XBX-1 is used to mark the basal body-transition fibres and axoneme. Scale bar, 4 μm. (**E**) Dye-filling assays, which probe for intact ciliary structures, reveal a genetic (synthetic) interaction between *cep-290* and an NPHP module mutant (*nphp-4*; dye-filling lost, shown as “-”) but not an MKS module mutant (*mks-2*; dye-filling normal, “+++”). Scale bar, 40 μm. (**F**) Schematics showing *cep-290* genetic interaction with *nphp-4* but not *mks-2* (top), and interdependent organisational hierarchy of MKS-5, CEP-290, and MKS and NPHP components (bottom). Together, the data point to CEP-290 functioning downstream of MKS-5 and upstream of MKS module components.

Having confirmed that CEP-290 is important for the formation of the TZ and function of the ciliary gate, we tested for its potential role in localising MKS module components (including TMEM-218) as well as NPHP module proteins and MKS-5 at the TZ. We first observed that TMEM-218 is consistently absent from the TZ in the *cep-290* mutant (Fig 4A). Moreover, four additional MKSome components, namely, the “core” TZ proteins MKSR-1 (mammalian B9d1), MKS-2 (Tmem216), and TMEM-231 (Tmem231), as well as TMEM-17 (Tmem17), are all mislocalised in the *cep-290* mutant (Figs 3C and 4A). Notably, how the proteins are mislocalised may be different (e.g., MKS-2 “leaks'”into the axoneme, whereas TMEM-17 remains in the dendrite; Figs 3C and 4A); the reason for this is unclear.

In contrast, we find that MKS-5 remains largely within the proximal region of *cep-290* mutant cilia in the area where the TZ would normally form (Fig 4A). This suggests that while both CEP-290 and MKS-5 play critical roles in the assembly of MKS module proteins at the TZ, MKS-5 can localise independently of CEP-290 and, therefore, may function “upstream” of CEP-290 during TZ assembly. We note that the localisation of MKS-5 in the *cep-290* mutant is not entirely wild-type, as it is in some cases more spread out and present within a more distal region of the axoneme (Fig 4A). This suggests that CEP-290 may provide additional stability and/or specificity to MKS-5 localisation at the TZ. In addition, both NPHP module proteins (NPHP-1 and NPHP-4) remain associated with the proximal region of the axoneme in the *cep-290* mutant, in a manner that is very similar to MKS-5 (Fig 4A; note that NPHP-1/NPHP-4 also localises to the basal body-transition fibres, as shown in Jensen et al. [30]). Hence, CEP-290 is required for the localisation of MKS module components but is not essential for the largely normal TZ localisation of the NPHP module or MKS-5. As we elaborate in the discussion, this result is both striking and surprising: in the absence of CEP-290, both MKS-5 and NPHP module proteins are present at the base of the cilium, in a region where the TZ is expected, even though no TZ ultrastructure (Y-links) is present (Fig 3E).

We performed reciprocal localisation experiments to ascertain if CEP-290 itself depends on other TZ proteins for its localisation. We show that CEP-290 is correctly localised in the *tmem-218* mutant and the core MKS module TZ mutant, *mks-2* (Fig 4B). Similarly, CEP-290 localisation is not affected by disruption of the core NPHP module protein, NPHP-4 (Fig 4B). In contrast, CEP-290 localisation to the TZ is lost when the core assembly factor, MKS-5, is ablated (Fig 4B). Specifically, the CEP-290::GFP signal is significantly reduced in the absence of MKS-5 and is often undetectable at the distal end of the dendrite despite the lower but not highly different *cep-290* transcript levels between wild-type and *mks-5* mutant (approximately 50% lower); notably, expression of *cep-290* in the *mks-2* mutant is slightly lower than in the *mks-5* mutant, yet localisation of CEP-290 is unperturbed (Figs 4B, 4D and S1B). This suggests that, in the absence of MKS-5, the CEP-290 protein is not incorporated into to the TZ region, may not be transported to the dendritic tip, and/or may be degraded. Furthermore, of the remaining CEP-290 observable near the ciliary region, the signal is closer to the basal body-transition fibers compared to its TZ localisation in wild-type animals (Fig 4C); it is also significantly lower in intensity and occupies a smaller volume (S1C Fig). Given the limiting resolving power of confocal microscopy, our results suggest that, in the absence of MKS-5, residual CEP-290 can localise to the very distal end of the dendrite but is not incorporated within the proximal region of the cilium.

To ascertain how MKS-5 might enable CEP-290 localisation to the TZ, we queried whether the coiled coil N-terminal domain of MKS-5 alone could confer this activity. Notably, the coiled coil region is both necessary and sufficient for MKS-5 localisation to the TZ, independent of its two C2 and RPGR-interacting domain regions [30]. We expressed the MKS-5 coiled coil region in the *mks-5* mutant, and found that it fully rescues CEP-290 localisation at the TZ, similar to full-length MKS-5 (Fig 4D). Furthermore, the MKS-5 coiled coil region restores the space (volume) normally occupied by CEP-290 at the TZ (S1C Fig).

We then sought to uncover if *cep-290* is genetically associated with the MKS module using genetic interaction and dye-filling studies. The *cep-290* mutant is able to dye-fill normally (Fig 4E), as expected from having largely intact, environmentally-exposed ciliary axonemes (Fig 3E). A synthetic dye-filling phenotype is observed when *cep-290* is combined with the *nphp-4* mutant, but not with the *mks-2* mutant (Fig 4E); this behaviour mimics that of other MKS module genes (e.g., *mksr-1*, *mks-2*, and *mks-6*). Rescue of the *cep-290* mutant with our CEP-290::GFP translational reporter confirms that *cep-290* is responsible for this phenotype and that the GFP-tagged protein is functional—making its TZ localisation biologically relevant (S2 Fig). Genetically, *cep-290* is, therefore, aligned with the MKS module of TZ genes (Fig 4F). Together, our findings are consistent with a hierarchical organisation at the TZ, wherein MKS-5 is positioned at the very “base,” influencing both NPHP and MKS module assembly, and CEP-290 is positioned between MKS-5 and MKS module components (Fig 4F).

### CDKL-1 and TMEM-138 Assembly at the TZ Requires CEP-290 but Is Independent of Core MKS Module Proteins

**Fig 5:**
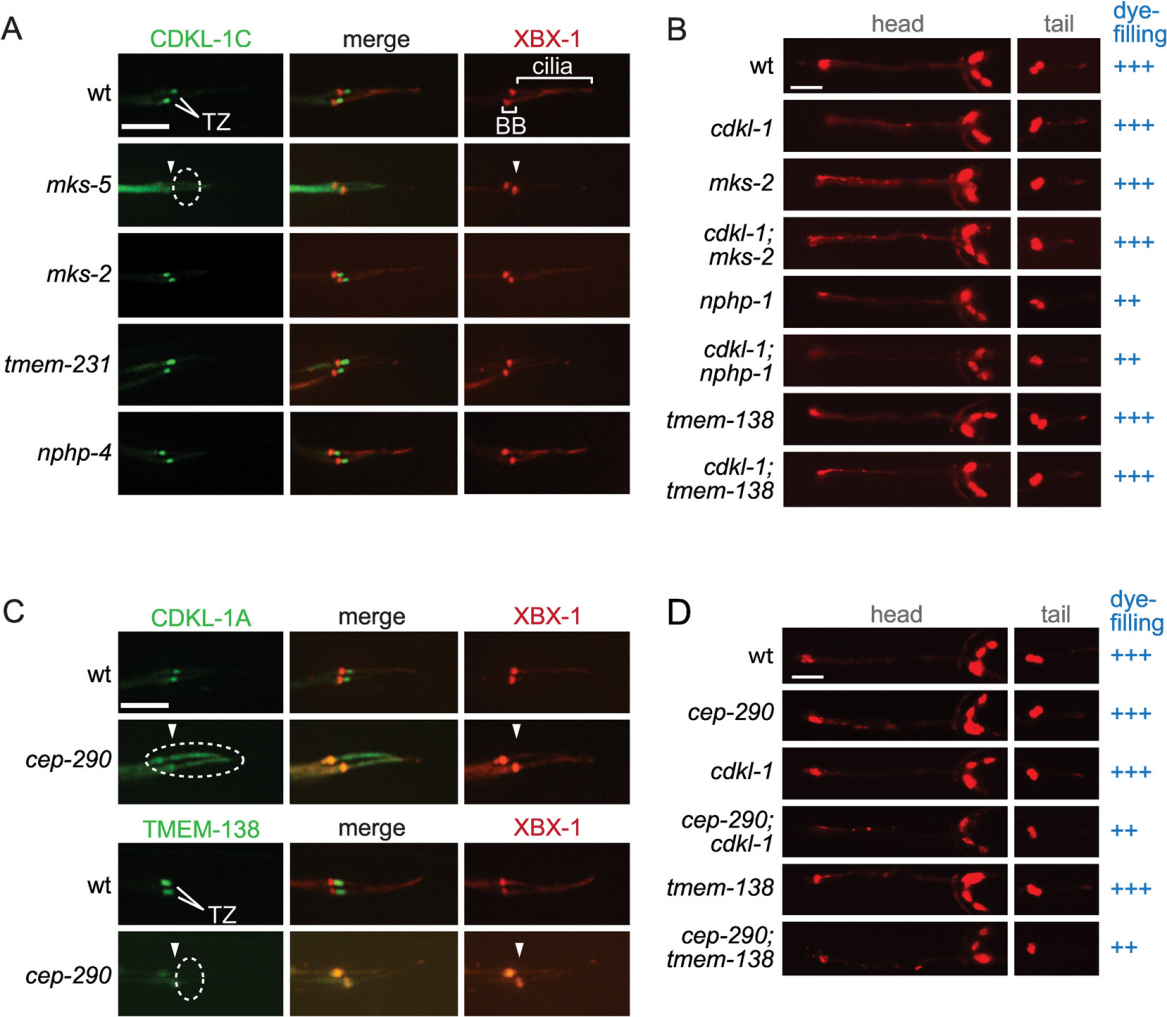
CDKL-1 and TMEM-138 require CEP-290 for their transition zone localisation and are functionally independent of the MKS or NPHP module. (**A**) GFP-tagged CDKL-1 (green) requires MKS-5 (dotted ellipse) but not other “core” MKS (MKS-2, TMEM-231) or NPHP (NPHP-4) module proteins for its TZ localisation. XBX-1 is used to mark the basal body-transition fibres and axonemes. Scale bar, 4 μm. (**B**) Dye-filling assays of head (amphid) and tail (phasmid) sensory neurons reveal that *cdkl-1* does not genetically interact (i.e., shows synthetic dye-filling phenotype) with either MKS (*mks-2*) or NPHP (*nphp-1*) module mutants. *cdkl-1* also does not show a genetic interaction with *tmem-138*. Scale bar, 40 μm. (**C**) Both GFP-tagged CDKL-1 and TMEM-138 depend on CEP-290 for their localisation to the TZ (dotted ellipse). Scale bar, 4 μm. **(D)** Partial (weak) dye-filling phenotype in the *cep-290;tmem-138* and *cep-290;cdkl-1* double mutants (++) compared to wild-type and the individual mutants (normal dye-filling, +++).

In *C*. *elegans*, all TZ proteins tested fit genetically within the MKS or NPHP module, the exception being the central organising or assembly factor, MKS-5, which shows interactions with both modules [25,28,30]. Here, we show that two proteins (CDKL-1 and TMEM-138) that require MKS-5 for TZ localisation and do not behave as either MKS or NPHP module proteins depend on CEP-290 for their correct localisation. The *C*. *elegans* CDKL-1 kinase, homologous to mammalian Cdkl proteins (Cdkl1/Cdkl2/Cdkl3/Cdkl4), localises to the TZ and influences cilium length, similar to the recently described *Chlamydomonas* CDKL5 protein (manuscript in preparation) [58]. We find that CDKL-1 (isoform A or C) is still TZ-localised when core MKS or NPHP module proteins are removed (Fig 5A). Furthermore, combining the *cdkl-1* mutant with either the *mks-2* mutant or the *nphp-1* mutant does not cause a synthetic dye-filling defect (Fig 5B). Hence, CDKL-1 cannot be assigned to either the MKS or NPHP module. Remarkably, however, CDKL-1 not only requires MKS-5 for its TZ localisation but also depends on CEP-290 (Fig 5C).

To determine if additional TZ proteins exhibit this behaviour, we examined TMEM-138, the *C*. *elegans* orthologue of mammalian Tmem138 [59]. Similar to CDKL-1, TMEM-138 requires MKS-5 for its localisation but does not depend on either core MKS or NPHP module proteins; furthermore, the *tmem-138* mutant shows no synthetic dye-filling defect with *mksr-1* or *nphp-4*, making an assignment to either module not feasible [30]. Remarkably, TMEM-138 also depends on CEP-290 for its TZ localisation (Fig 5C). Together, our findings suggest hierarchical/functional characteristics for CDKL-1 and TMEM-138 that differ from all other MKS module proteins but that share a dependence on both MKS-5 and CEP-290 for their TZ localisation. For the purpose of discussion below, we refer to CDKL-1 and TMEM-138 as CEP-290-associated proteins. We wondered if the two genes interact genetically, but the *tmem-138;cdkl-1* double mutant does not show a synthetic ciliogenesis (dye-filling) phenotype (Fig 5B). We further queried if there are genetic interaction(s) between *cep-290* and *tmem-138* and/or *cdkl-1*. Only a small difference in dye-filling (weaker uptake) was found between the double mutants (*tmem-138;cep-290* and *cdkl-1;cep-290*) compared to the single mutants, consistent with the positioning of *tmem-138*, *cdkl-1*, and *cep-290* in the same genetic module (Fig 5D).

### Evidence for Expanded Role for TZ Proteins in Ciliopathies and Ciliogenesis

**Fig 6:**
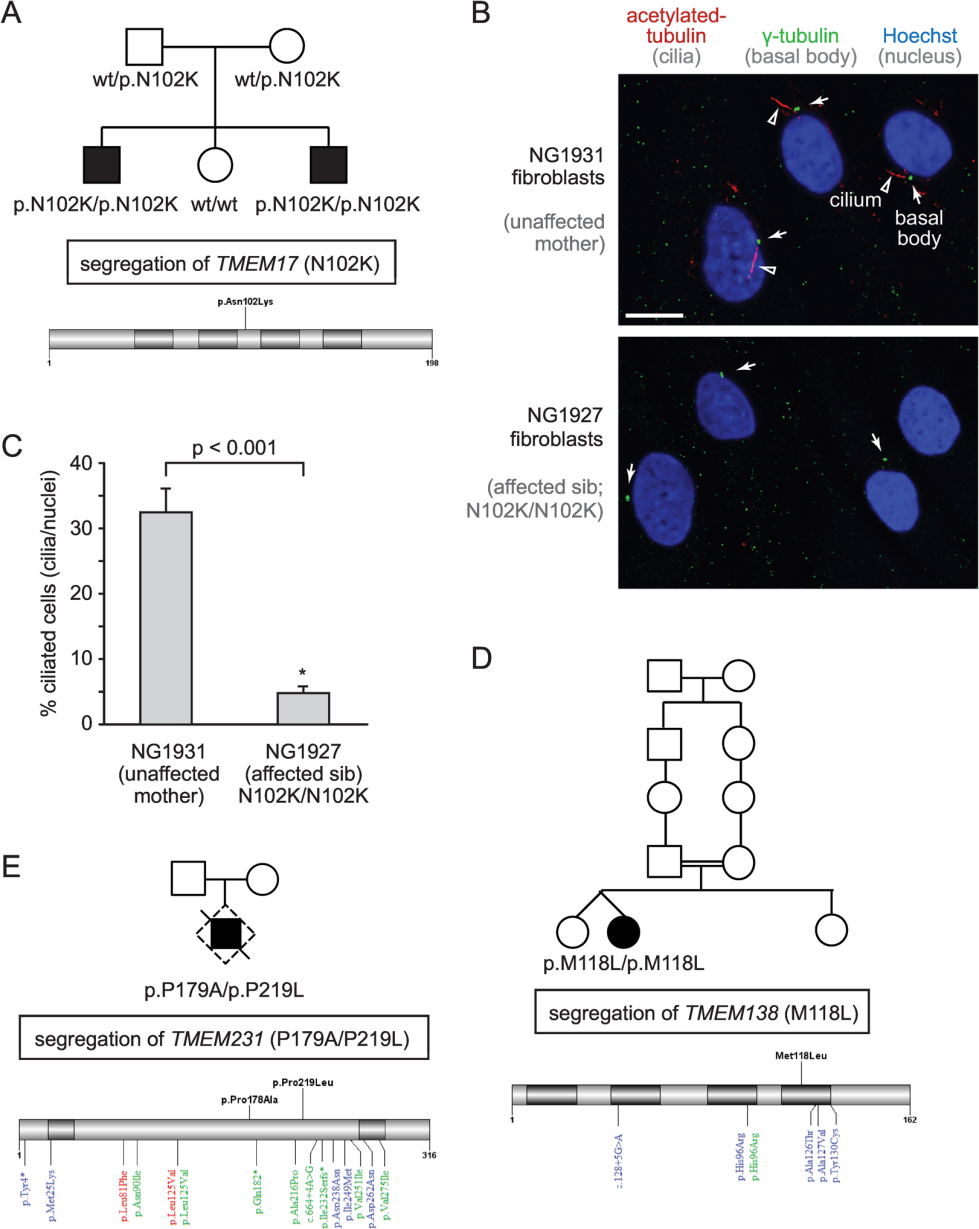
Potential expanded role for TZ proteins in ciliopathies and ciliogenesis: Tmem17, Tmem138, and Tmem231 are mutated in Oral-Facial-Digital type 6 (OFD6) syndrome families, and Tmem17 mutant fibroblast cells display ciliogenesis defects. (**A**) Pedigree of OFD6-affected individuals with a recessive missense mutation in *TMEM17* that affects a conserved amino acid (p.N102K). Schematic displays the position of the mutation within the *TMEM17* gene (NM_198276). (**B**) Fluorescence images of fibroblasts reveals full-length cilia in a healthy control but significantly fewer cilia in the OFD6-affected patient. Cilia and basal bodies are detected with antibodies against acetylated tubulin (arrowheads; red) and γ-tubulin (arrow; green), respectively, and nuclei are stained with Hoechst (blue). Scale bar, 10 μm. (**C**) Quantitation showing a ciliogenesis defect (cilia/nuclei) in Tmem17 (p.N102K/p.N102K)-mutant fibroblast cells compared to the unaffected control. Statistical significance inferred from Student’s *t* test. (**D, E**) Family pedigrees of OFD6-affected individual with a recessive homozygous mutation in *TMEM138* (p.M118L) and compound heterozygous composite mutations in *TMEM231* (p.P179A/p.P219L). Schematics of *TMEM138* (NM_016464.4) and *TMEM231* (NM_001077416.2) gene structures, with previously identified mutations implicated in Oral-Facial-Digital type 3 (red), Joubert (blue), and Meckel (green) syndromes (bottom); new mutations are shown on top (black).

The majority of known TZ proteins are associated with one or more ciliopathies, including MKS, JBTS, SLNS, and NPHP [21,22,26]. Our recent discovery that *C*. *elegans* TMEM-17 is an MKS module protein [30] that depends on CEP-290 for TZ localisation (Fig 4A) suggests that it may also be linked to one or more ciliopathies; however, such a possibility has not been reported. We therefore included *TMEM17* in a panel of 101 ciliary genes (S1 Table) that underwent next-generation sequencing (NGS) in a cohort of 330 patients with a neuroradiologically confirmed diagnosis of JBTS and variable organ involvement (S2 Table). Interestingly, we uncovered a homozygous missense mutation in *TMEM17* (p.N102K) in two siblings whose clinical profile is consistent with OFD6 (Figs 6A and S3A), a subtype of JBTS [60].

The *TMEM17* variation fully segregates with the disease in the family (Figs 6A and S3A) and is not detected in 150 in-house controls or in over 73,000 samples from the public databases EXAC and EVS. Furthermore, the p.N102K variant is predicted to be pathogenic by four distinct bioinformatic predictors (PolyPhen2, SIFT, Mutation Assessor, Mutation Taster) and affects a highly conserved amino acid residue (S3B Fig). Importantly, we show that fibroblast cells isolated from a *TMEM17-*mutated sibling display a much reduced ability to form cilia compared to cells obtained from the healthy heterozygous mother (Fig 6B and 6C). Together, the discovery of a homozygous mutation in a highly conserved residue of Tmem17 that is not present in control genomes and is correlated with a defect in ciliogenesis provides strong evidence that disruption of Tmem17 results in a ciliopathy, OFD6.

Notably, the *C5orf42* gene was previously thought to be the main cause of OFD6 [61], although this is now debated [62]; and a second gene related to *TMEM17*—*TMEM216* (*MKS2*)—has also been associated with OFD6 [63]. We therefore sought to identify additional genes that encode CEP-290-dependent TZ proteins that may be mutated in OFD6 patients. We identified individual families with missense mutations in two genes, *TMEM138* (homozygous p.M118L) and *TMEM231* (compound heterozygous p.P179A/p.P219L) (Figs 6D, 6E and S4; S2 Table). All mutant alleles are predicted to be pathogenic in that they affect conserved residues, are not found in control cohorts or public databases, and fully segregate with the disease in the respective families (Fig 6D and 6E).

Collectively, our findings provide strong evidence that Tmem17 is a novel OFD6-associated protein that is necessary for ciliogenesis and suggest a potentially expanded spectrum of TZ-associated proteins (Tmem138, Tmem231) linked to this JBTS phenotype. Since not all genes linked to TZ-associated ciliopathies have been uncovered, we speculated that other TZ-associated ciliopathy proteins likely exist. Indeed, the Tmem17/Tmem216-related protein that emerged in tetrapods (S5A Fig), Tmem80, also represented an excellent, uncharacterised candidate. Using immunofluorescence analysis of wild-type and TZ mutant mouse embryonic fibroblasts, we show that mammalian Tmem80 localises to the TZ in a manner dependent on MKS module-associated proteins (Cc2d2a/Mks6, Tctn1, and Tctn2) (S5B Fig). Although we sequenced *TMEM80* (as well as *TMEM218*) in our patient cohort and did not find any pathogenic variants (S1 Table), we propose that both genes represent excellent candidates for being associated with OFD6 or other related ciliopathies, including MKS and JBTS.

## Discussion

**Fig 7:**
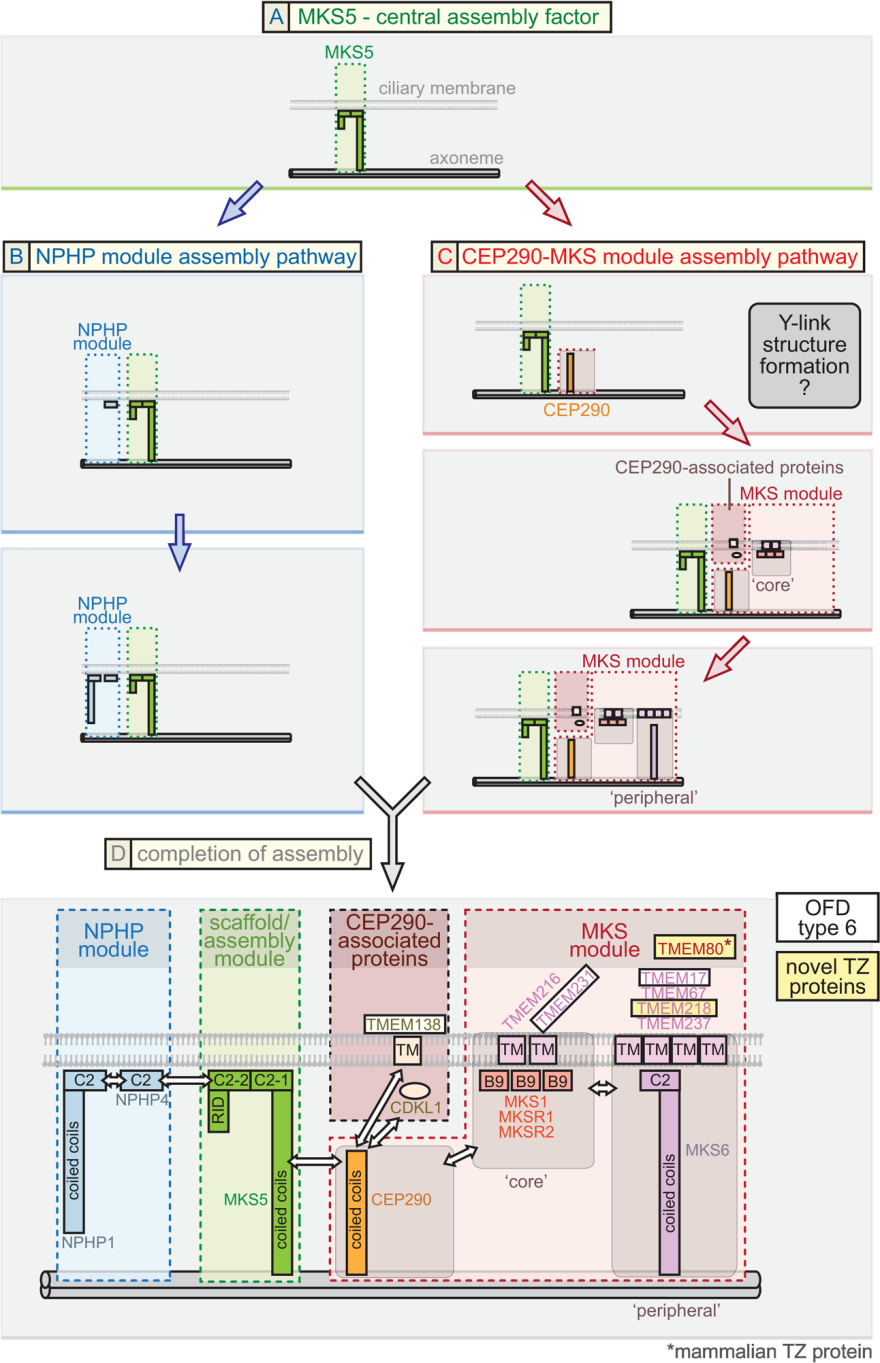
Proposed model for ciliary transition zone assembly. This model for the assembly of proteins at the TZ is based mostly on *C*. *elegans* data but is largely consistent with findings from other organisms, including mammals, *Chlamydomonas*, and *Drosophila*. (**A**) **MKS5: central assembly factor**. *C*. *elegans* MKS-5 (mammalian Rprgrip1L/Rpgrip1 orthologue) is essential for the assembly of all known TZ proteins (including CEP-290) and, therefore, may be the first component to establish the TZ. MKS-5 directs two genetically separable (distinct) assembly pathways, involving NPHP module proteins (blue arrows) as well as CEP-290, CEP-290-associated proteins, and MKS module proteins (red arrows). (**B**) **NPHP module assembly pathway**. Results from this study, other *C*. *elegans* findings, and mammalian work suggest that NPHP module proteins function together with MKS-5. We propose that the NPHP module, which can assemble at the TZ in the absence of CEP-290 (red pathway), assembles either before or in conjunction with the CEP-290-dependent pathway. NPHP-1 requires NPHP-4 for assembly at the TZ and is, therefore, positioned “downstream” in the pathway. (**C**) **CEP290-MKS module assembly pathway**. CEP-290 depends on MKS-5 for its TZ localisation and is, therefore, shown to assemble “downstream” in the pathway. Established MKS module proteins, including “core” components, require CEP-290 for TZ localization and are shown to assemble “downstream.” Another subset of TZ proteins (CDKL-1, TMEM-138) depends on CEP-290, but not MKS module proteins, for TZ localization (termed “CEP290-associated proteins”); their assembly is tentatively shown to occur simultaneously with the “core” MKS module components. TZ ultrastructure (Y-links) can be observed when “core” MKS or NPHP module proteins are disrupted, but not when CEP-290 is removed, and so we tentatively assign the formation of the Y-links at the CEP-290 assembly stage. More “peripheral” MKS module components require “core” MKS module components for TZ localisation and, hence, are proposed to assemble “downstream” of the core components. CEP-290, CEP-290-associated proteins, and MKS module (“core” and “peripheral”) proteins are shown being incorporated at distinct steps of an assembly process, but the possibility of pre-assembly exists; for example, “core” MKS module proteins might pre-assemble prior to incorporation into the TZ, and “peripheral” proteins might interact with the “core” proteins prior to the assembly stage shown. (**D**) **Completion of TZ assembly and link to the ciliopathy OFD6**. MKS-5, the NPHP module, and the CEP-290-MKS module all combine to yield the complete TZ. A detailed spatial/topological/hierarchical map of TZ proteins, relative to the axoneme (double cylinders at bottom) and ciliary membrane (bilayer shown), is suggested. Pertinent domains shown in the TZ proteins include membrane-associated C2 and related B9 domains, transmembrane (TMEM) domains, and coiled coils. Major connections (potentially direct or indirect) between different modules or proteins are depicted with double-headed arrows and based on a combination of data from *C*. *elegans* and other organisms. Novel TZ proteins we uncovered in this study (*C*. *elegans* TMEM-218 and mammalian Tmem80) and proteins we provide evidence for being associated with OFD6 (Tmem17, Tmem138, Tmem231), are highlighted in yellow and white boxes, respectively.

The TZ represents a major, evolutionarily conserved domain at the base of cilia whose supramolecular organisation and mechanism of function as a selective membrane diffusion barrier remains largely unknown. Our findings uncover *C*. *elegans* CEP-290 as an essential assembly factor for the genetically and biochemically defined “MKS” functional module. Loss of CEP-290 abrogates the TZ localisation of MKS module components, including that of a novel TZ protein we discovered, TMEM-218. Our genetic, cell biology, and in vivo functional assays reveal a critical role for CEP-290 in formation of the membrane diffusion barrier. Furthermore, the findings reported here—combined with our previous work and consistent with proteomic analyses—allow us to propose a plausible, comprehensive model for the assembly pathway of the TZ. In this model, MKS-5 (Rpgrip1L/Rpgrip1 orthologue) represents the foundation of two distinct assembly pathways for the MKS and NPHP modules, with the MKS pathway having CEP-290 as its core (Fig 7A).

### MKS-5-Dependent Pathway for the Assembly of NPHP Transition Zone Module Components

One branch of the MKS-5-dependent pathway involves the assembly of the NPHP module, consisting of NPHP-1 and NPHP-4 (Fig 7B). Our work reveals that its assembly at the TZ can occur independently of CEP-290. Notably, when CEP-290 is disrupted, both NPHP-1 and NPHP-4, as well as MKS-5, colocalise within the proximal region of cilia, where the TZ is normally found (Fig 4A). This represents evidence for a functional association between the NPHP-1/NPHP-4 and MKS-5 proteins, consistent with the discovery by Sang and colleagues that mammalian Nphp1/Nphp4 proteins are observed in a complex with Mks5 (Nphp8/Rpgrip1L) [39]. Our results also suggest the possibility that MKS-5 together with NPHP-1/NPHP-4 represent the first components to establish a region at the base of cilia that give rise to the canonical TZ. Indeed, this MKS-5-NPHP-1-NPHP-4 domain is established in the *cep-290* mutant, which has no discernible TZ ultrastructure, including Y-links (Fig 3E). Given these findings, we speculate that MKS-5 and the NPHP module might help nucleate the assembly of Y-links—whose structural makeup may include CEP-290 and, perhaps, other associated proteins—as well as establish the position and appropriate length of the TZ. Hence, in the *cep-290* mutant, Y-links are absent, but the ciliary axoneme “foundation” for a TZ remains. Such an intriguing possibility will require further confirmation.

### MKS-5 and CEP-290-Dependent Pathway for the Assembly of MKS Module Transition Zone Components

The second branch of the MKS-5-dependent pathway first involves the assembly of CEP-290 (Fig 7C, first step) and then MKS module components, including “core” and “peripheral” TZ proteins, which are all predicted to be transmembrane proteins or have membrane associations via C2 or B9 domains (Fig 7C). Notably, *C*. *elegans* CEP-290 is specifically required for MKS module protein assembly, as it is not needed for NPHP module protein assembly at the TZ (Figs 3C and 4A). This is consistent with the finding that in *Drosophila*, NPHP module proteins are not present and Cep290 is required for the correct localisation of B9-domain MKS module proteins (Mks1, B9d1, and B9d2) [47]. Pull-down studies in mammalian cells are similarly consistent with an association between Cep290 and various MKS module proteins [40,64]. Also consistent with the above, Nphp4 is TZ-localised in the *Chlamydomonas* Cep290 mutant [46]. Finally, we observe a genetic interaction between *cep-290* and an NPHP module gene (*nphp-4*) that is required for ciliogenesis, but no interaction between *cep-290* and an MKS module gene (*mks-2*) (Fig 4E and 4F), precisely as with all known MKS module genes [25,28,31].

Given these findings, we propose that CEP-290 is specifically required for the assembly of the entire MKS module. Since MKS-1/MKSR-1/MKSR-2/MKS-2/TMEM-231 all depend on each other for their TZ localisation [25,28,36], we propose that these are “core” TZ proteins that assemble “downstream” of CEP-290 (Fig 7C, second step). This hierarchical definition implies that TMEM-17, TMEM-67 (MKS-3), TMEM-218, and TMEM-237 are more “peripheral,” as they fail to assemble at the TZ when any “core” TZ protein is disrupted and do not themselves influence the localisation of the “core” TZ proteins (this study and refs. [25,28,30]). Assembly of “peripheral” TZ proteins is likely to be “downstream” of “core” TZ proteins (Fig 7C, third step). Together with these localisation studies, a genetic interaction between *cep-290* and a core NPHP module mutant (*nphp-4*), but not a core MKS module mutant (*mks-2*), argues for a clear positioning of CEP-290 as the core component of the MKS module (Fig 7C). Notably, our hierarchy for *C*. *elegans* CEP-290 differs from that of Schouteden and colleagues [65], who propose that the TZ protein occupies an entirely different (third) module.

### CDKL-1 and TMEM-138 Are Assembled at the TZ in a CEP-290-Dependent and MKS Module-Independent Manner

Aside from MKS-5, which is the core assembly factor, all *C*. *elegans* TZ-localised proteins analysed heretofore can be assigned to an MKS or NPHP module. Here, we show that CDKL-1 (a novel TZ protein homologous to uncharacterised human CDKL family members) and TMEM-138 (whose human orthologue Tmem138 is mutated in JBTS [59]) both require CEP-290 for their TZ localisation (Fig 5C). Given our above-mentioned findings, this might suggest an association between CDKL-1/TMEM-138 and the MKS module. Intriguingly, however, neither protein is delocalised when core MKS module proteins are disrupted (see Fig 5A and Jensen et al. [30]). Moreover, the *tmem-138* and *cdkl-1* genes do not behave like MKS module genes, in that their respective mutants do not show a synthetic ciliogenesis defect with NPHP genes (Fig 5B and [30]). Altogether, these findings suggest that CEP-290 may itself organise two “branches” of an extended MKS module: one that harbours “canonical” MKS module proteins and another that consists of CDKL-1, TMEM-138, and, perhaps, additional TZ proteins (Fig 7D). Interestingly, CDKL-1 does not appear to influence cilium gate function but does influence cilium length (manuscript in preparation), the latter being similar to its CDKL5 homologues in *Chlamydomonas* [58,66]. Thus, CEP-290 may help organise a novel module that may be hierarchically and functionally different from the well-established NPHP and MKS modules. Interestingly, Lee and colleagues [59] provided some insights into possible behavioural differences between Tmem138 and Mks2, including with respect to Cep290. The Cdkl1/Cdkl2/Cdkl3/Cdkl4 family of kinases is largely unstudied, but a zebrafish knockdown study of Cdkl1 suggests ciliary-like phenotypes (including abrogated Hedgehog signalling) [67].

### CEP-290 Is Required for Ciliary Gate Function and Assembly of Y-Links

Having ascertained that CEP-290 plays a central role in the assembly of the MKS module, we confirmed that it is also essential for the formation of a functional ciliary gate. Specifically, loss of CEP-290 causes two proteins (TRAM-1a and RPI-2) normally found at the periciliary membrane (just outside of the cilium) to accumulate abnormally within the organelle (Fig 3C). This “leakiness” has been observed with most MKS and NPHP module mutants and is also consistent with changes to ciliary composition in the *Chlamydomonas* Cep290 mutant [48]. Furthermore, we show that ARL-13 enters the TZ and accumulates at the periciliary membrane in the *cep-290* mutant (Fig 3D), which represents additional evidence that CEP-290 helps form a functional TZ membrane diffusion barrier.

Previous TEM analyses of *C*. *elegans* TZ gene mutants revealed that disruption of one or more MKS module component(s), or NPHP module component(s), does not abrogate the formation of Y-links (with the exception of *nphp-4*, which has modest defects [29]). However, various MKS-NPHP double mutants, such as *mks-2;nphp-4* or *mksr-1;nphp-4*, result in the apparently complete loss of Y-shaped structures [25,28,36]. Here we show that disruption of CEP-290 alone is sufficient to eliminate Y-links (Fig 3E). This *cep-290* phenotype is striking in that it influences to a great extent only the TZ region, wherein Y-links are absent. Yet, as discussed above, in the *cep-290* mutant, the most “central” assembly factor, MKS-5, together with the NPHP module, are present at the base of the cilium, in the same position expected for the TZ. Hence, removal of MKS-5 may cause a loss of Y-link structures [30], potentially because CEP-290 itself cannot assemble at the TZ.

Based on this reasoning, we suggest in our model for the assembly pathway of the TZ that Y-link structures are established at the step at which CEP-290 assembles at the TZ (Fig 7C, first step). This inference is based on the fact that Y-link formation is not impaired when one or more “core” or “peripheral” MKS module proteins are disrupted, which likely results in all MKS module proteins being mislocalised [25,28,30,36]. Although the MKS module proteins are likely a part of the Y-links, they are all membrane-associated (using transmembrane of C2/B9 domains) and, therefore, may be more closely linked to the formation of the ciliary necklace (intramembraneous “beads” [68]) rather than the central/prominent region of the Y-shaped structure. We speculate that although Y-links are missing in the *C*. *elegans* TZ of the *cep-290* mutant, similar to that found by Schouteden et al. [65] and in the *Chlamydomonas* Cep290 mutant [48], structural proteins other than CEP-290 might make up these prominent Y-shaped structures.

### Tmem218 and Tmem80 Represent Novel TZ Proteins that May Be Ciliopathy-Associated

Virtually all genes encoding TZ proteins are linked to one or more ciliopathies, namely MKS, JBTS, SLNS, Leber Congenital Amaurosis, and NPHP [21,22,26,27]. Recently, disruption of Tmem218 in a mouse mutagenesis screen was found to cause retinal degeneration and NPHP phenotypes that resemble SLNS [50]. While the murine protein was not characterised, we show that the nematode orthologue (TMEM-218) localises to the TZ, as with other ciliopathy-associated TZ proteins (Fig 1B). Furthermore, TMEM-218 can be positioned genetically and hierarchically within the MKS module, likely as a peripheral component, similar to MKS-3 (Tmem67), JBTS-14 (Tmem237), and MKS-6 (Cc2d2a), which are all ciliopathy-associated (Figs 1D, 2D and 7). In addition, we show for the first time that the unchracterised Tmem17/Tmem216-related mammalian protein, Tmem80, localises to the TZ in ciliated mouse embryonic fibroblasts (S5 Fig). Notably, Tmem80 depends on at least three known TZ-localised ciliopathy proteins (Tctn1, Tctn2, and Mks6/Cc2d2a) for its localisation, positioning it within a growing functional network of TZ proteins.

Hence, both Tmem218 and Tmem80 represent prime candidates for being implicated in one or more of the above ciliopathies or OFD6, as discussed below. Although sequencing of *TMEM80* and *TMEM218* in a large cohort of patients with JBTS-related phenotypes did not identify pathogenic mutations, the possibility that such mutations represent a rare cause of OFD6 cannot be excluded at present, especially since the number of OFD6 patients in our cohort is quite low due to the rarity of this condition (17 probands).

### CEP290-Dependent Proteins May Be Linked to OFD6

Aside from Tmem218 and Tmem80, one of the few TZ proteins that remains to be linked to one or more ciliopathies is Tmem17. Here, we present evidence that a variation in an evolutionarily conserved residue of Tmem17 causes OFD6 syndrome. The mutation (N102K), not present in control cohorts, is predicted to be deleterious and segregates specifically with affected siblings in the family (Fig 6A). Importantly, we show that patient fibroblast cells with the pN102K mutation display a prominent ciliogenesis defect compared to control cells (Fig 6B and 6C). Notably, many TZ proteins, including B9d1, Tmem67/Mks3, Tmem231, Tmem237, Cc2d2a/Mks6, Cep290, and Tectonics (Tctn1 and Tctn2) are known to influence ciliogenesis in mammalian cells [28,38,40,69], consistent with our findings. Although only present in single families, we also provide evidence that the human orthologues of two additional proteins that require CEP-290 for their TZ localisation in *C*. *elegans*, Tmem138 and Tmem231, are also mutated in OFD6 (Figs 6D, 6E and S4). Uncovering additional families with mutations in *TMEM17*, *TMEM138*, and *TMEM231* will be important to conclusively assign these genes to OFD6. Finally, what TZ/ciliary defect may result in OFD6 rather than other related ciliopathies, including OFD3, remains unclear. For example, Tmem231 is linked to OFD3 as well as MKS [31].

### Conclusions

Understanding what components make up the TZ and how they assemble into different functional modules represent important goals for shedding light on how the TZ forms during ciliogenesis and acts as a membrane diffusion barrier. In addition to uncovering *C*. *elegans* TMEM-218 and mammalian Tmem80 as novel TZ proteins, our work illuminates the central roles of MKS-5 and CEP-290 in the assembly of NPHP and MKS modules, and of TZ ultrastructure (Fig 7). Aside from CEP-290, which proteins constitute the Y-links remains an open question. How at least two TZ proteins (CDKL-1 and TMEM-138) assemble in a CEP-290-dependent but MKS module-independent manner is unclear, but hints at the existence of a TZ module that is distinct from the MKS and NPHP modules. It will be interesting to confirm and further investigate in mammalian cells our overall model for TZ assembly (Fig 7) as well as obtain evidence for the involvement of the novel TZ proteins we uncovered (Tmem218 and Tmem80) in ciliopathies.

## Materials and Methods

### Preparation of *C*. *elegans* Transgenic Constructs

The translational construct for the *cep-290* (Y47G6A.17) gene was generated by fusing the *bbs-8* promoter (941 bp) to the cDNA of *cep-290* and EGFP together with the *unc-54* 3’ UTR. The translational construct for *tmem-218* (T23E7.5) was generated by fusing its native promoter (2,092 bp upstream of the start codon) and all exons and introns to EGFP together with the *unc-54* 3’ UTR. For *cdkl-1* (Y42A5A.4), we generated two constructs. One was made by fusing the *bbs-8* promoter (941 bp) to the genomic region of *cdkl-1* (isoform C) and EGFP with the *unc-54* 3’ UTR. The other was generated by fusing its native promoter (1,869 bp) and all exons and introns (*cdkl-1* isoform A) to EGFP with the *unc-54* 3’ UTR. Transgenic lines were generated as reported previously [51].

### *C*. *elegans* Strain Maintenance, Construction, and Imaging

All *C*. *elegans* strains used in this study (S3 Table) were maintained and cultured at 20°C. Those carrying mutations in *C*. *elegans mks-5(tm3100)*, *tmem-231(tm5963)*, *cdkl-1(tm4182)*, *cep-290(gk415029)*, *tmem-138(tm5624)*, *nphp-1(ok500)*, and *nphp-4(tm925)* were obtained from the *C*. *elegans* Gene Knockout Consortium or National Bioresource Project and outcrossed to wild-type (N2) a minimum of five times. Standard mating procedures were used to introduce GFP-tagged protein constructs into different genetic backgrounds. Genotyping the various mutants was done by single-worm PCR. Confocal microscopy was used to assess the subcellular localisation of the various fluorescent reporters for both the wild-type and each TZ mutant strain, as indicated. A minimum of 50 animals for each strain were analysed for all reported mislocalisation phenotype. We obtained a *cep-290(gk415029)* allele, which has a G→A nonsense mutation at nucleotide 3989 in the *cep-290* gene. The *gk415029* allele is likely to be null, as it contains a stop codon 36.7% into the coding region.

The mutagenesis protocol for generating a *tmem-218* (T23E7.5) allele was modified from Huang et al. (2011) using the ttTi20388 allele [70], which contains a Mos1 insertion in the intron of *tmem-218*. Sequencing of the *tmem-218(nx114)* allele revealed a 2721 bp deletion plus 4 bp insertion that removes the entire *tmem-218* coding region. Before analysis, *tmem-218(nx114)* worms were outcrossed six times to wild type (N2).

### *C*. *elegans* Dye-Filling Assay for Assessing Intact Ciliary Structure

*C*. *elegans* were exposed to the lipophilic dye, DiI, to assay for uptake of dye through intact, environmentally exposed ciliary structures, as previously described [25]. Stained worms were imaged with fluorescence microscopy, and intensities were analysed with ImageJ.

### Transmission Electron Microscopy of *C*. *elegans* Amphid Cilia

Young adult worms were fixed, sectioned, and imaged as described previously [71], with the exception that worms were fixed overnight at 4°C in 2.5% gluteraldehyde, 1% paraformaldehyde in Sørensen's buffer.

### Analysis of CEP-290 Subcellular Localisation

CEP-290::GFP localisation in wild-type or *mks-5* mutant animals was assessed relative to the position of the basal body-transition fibres marked by the XBX-1::tdTomato IFT protein marker. Distance (in μm) from the peak fluorescence value of XBX-1 (middle of the basal body-transition fibres) to the first CEP-290 pixel above 0.5 (relative intensity) is defined here as the start of the TZ. The size of the TZ is the length (in μm) from the first pixel of CEP-290 fluorescence over 0.5 to the last pixel over 0.5.

### CEP-290 Localisation Volume Analysis

All images were taken on a spinning-disc confocal of phasmid (PHA and PHB) cilia, as described above, with the same settings for the same exposure time (600 ms). CEP-290::GFP localisation volumes were detected by the Volocity software “find object” program with an automatic threshold offset of 200. *p*-values were calculated by Dunn Kruskal-Wallis multiple comparison (Holm-Sidak method).

### Quantification of Transgene Expression Levels in Phasmid Neurons

Anaesthetized L4 stage transgenic worms in 10 mM levamisole were prepared for spinning-disc confocal microscopy. Fluorescence in at least 20 pairs of cell bodies of PHA and PHB neurons from strains expressing TMEM-218::GFP, TMEM-231::GFP, or CDKL-1::GFP (isoforms A and C) was visualized and analyzed using Volocity software (Perkin Elmer). To obtain relative fluorescence intensity for each strain, the background fluorescence signal was subtracted and the absolute fluorescence intensity measurements were divided by the median fluorescence intensity in wild type. The relative fluorescence intensity data were plotted using Box and Dot plots in R. The assessment of normal distribution of each dataset was done using the Shapiro–Wilk test. The *p*-value of the dataset that shows normal distribution was obtained by Tukey multiple comparisons of means of 95% family-wise confidence level. The *p*-value of the dataset that shows non-normal distribution was calculated using the Kruskal-Wallis test; Dunn Kruskal-Wallis multiple comparison (Holm-Sidak method) was used for comparing more than two groups. Results are presented in S1D–S1G Fig.

Quantitative RT-PCR to measure CEP-290::GFP and *cep-290* transcript levels was performed as previously described [68] using iTaq Universal SYBR green supermix (Biorad), an Applied Biosystems StepOne real-time PCR system, SuperScript III reverse transcriptase (Invitrogen), and the following primers: control, *tba-1* forward: tcaacactgccatcgccgcc and reverse: tccaagcgagaccaggcttcag, GFP forward: atggtgttcaatgcttctcg and reverse: tgtagttcccgtcatctttga, and *cep-290* forward: tgctcagcgagttgaatagg and reverse: aagcttcccaatttgctcat.

### Screening of Ciliopathy Patients

The patient cohort included about 330 probands with JBTS, selected by the unique neuroimaging criterion of the molar tooth sign (MTS), including 17 probands with the OFD6 phenotype. For each patient, a standardised clinical questionnaire completed by the referring clinician provided detailed information on the phenotypic spectrum and organ involvement. Written informed consent was obtained from all families, and the study was approved by the local ethics committee.

All patients underwent simultaneous target sequencing of 100 genes (including genes known to cause human ciliopathies and candidate genes derived from functional studies) on a Solid 5500xL platform (Life Technologies). Probes were designed to cover all exons, with splice-site junctions and at least 25 bp of flanking introns. Bioinformatic analyses were conducted to filter data and remove low-quality calls as well as variants with a frequency of >1% in public databases dbSNP ver.141 (http://www.ncbi.nlm.nih.gov/SNP/) and Exome Variant Server (http://evs.gs.washington.edu/EVS/) or found in internal controls. The identified mutation in the *TMEM17* gene (NM_198276) was validated using bidirectional Sanger sequencing using the Big Dye Terminator chemistry (Life Technologies) and was searched against public databases dbSNP and Exome Variant Server. Potential pathogenicity was predicted using prediction software PolyPhen-2 ver.2.2.2 (http://genetics.bwh.harvard.edu/pph2/) and SIFT (http://sift.jcvi.org/). Nomenclature was assigned according to the Human Genome Variant Society (http://www.hgvs.org/mutnomen/). Sanger sequencing was also used to birectionally sequence all coding exons and exon–intron boundaries of *TMEM218* in a subset of 160 Joubert patients who had tested negative at the previous NGS experiment.

Probands from two large families diagnosed with OFD6 directly underwent whole exome sequencing. Three micrograms of genomic DNA per individual were subjected to whole-exome capture and sequencing using the SureSelect Human All Exon V5 kit (Agilent). The resulting libraries were sequenced on a HiSeq 2000 (Illumina) as paired-end 76 bp reads. BAM files were aligned to a human genome reference sequence (GRCh37/hg19) using BWA (Burrows-Wheeler Aligner; v0.6.2). All aligned read data were subject to the following steps: (1) duplicate paired-end reads were removed by Picard 1.77, and (2) indel realignment and (3) base quality score recalibration were done on Genome Analysis Toolkit (GATK; v2.1–10). Variants with a quality score >30 and alignment quality score >20 were annotated with SeattleSeq SNP Annotation (http://snp.gs.washington.edu/SeattleSeqAnnotation138/). Rare variants present at a frequency above 1% in dbSNP 138 (http://www.ncbi.nlm.nih.gov/SNP/) and the NHLBI GO Exome Sequencing Project or present from 312 exomes of unaffected individuals were excluded (http://evs.gs.washington.edu/EVS/). To improve exome analysis, data are crossreferenced with a list of known cilia-related genes from Ciliome Database, Cildb v2.1 (http://cildb-archive.cgm.cnrs-gif.fr/) and transcriptomic study of cilia [72]. The analyses were focused on genes with homozygous variants in consanguineous families and with two heterozygous variants in other cases, prioritising (1) genes associated with human pathology in ClinVar (http://www.ncbi.nlm.nih.gov/clinvar/) or HGMD databases (http://www.hgmd.cf.ac.uk/ac/index.php), (2) cilia-related genes, and (3) other non cilia-related genes. Candidate variants and parental segregation were confirmed by DNA PCR/Sanger sequencing using targeted primers (S5 and S6 Tables) and following usual protocols.

### Ciliogenesis Assays for Human Fibroblast Cells

Fibroblasts from one patient homozygous for *TMEM17* mutations and from his healthy mother (heterozygous carrier) were plated on coverslips and cultured in DMEM with 20% FBS until they were 80% confluent after being starved for 24 h in DMEM 0.1% FBS to allow cilia formation. Cells were fixed in cold methanol, and coverslips were rinsed and blocked in PBS with 10% BSA prior to incubation with antibodies. Fixed cells were incubated with the following antibodies: acetylated-α-tubulin (1:1000, SIGMA), γ-tubulin (1:500, SIGMA) overnight followed by incubation with goat anti-mouse Alexa fluor 555 (1:3000), and goat anti-rabbit Alexa fluor 488 (1:3000). Nuclei were stained with Hoechst (Invitrogen). Images were captured with a confocal microscopy (C2 Confocal Microscopy System), by using the laser lines 488 nm (green) or the 561 nm (red) and a 60X 1.4 NA Plan Apo objective (Nikon Corporation) controlled by NIS Element AR 4.13.04 software. To count cilia, acetylated-α-tubulin and γ-tubulin positive cilia were manually counted within 15 images for each phenotype in at least three independent experiments. Variables were analysed by Student’s *t* test, and a value of *p* < 0.05 was deemed statistically significant. Values are expressed as standard error (S.E.).

### Immunofluorescence Analyses in Mouse Embryonic Fibroblasts

Tctn1, Tctn2, Cc2d2a, Tmem67, B9d1, and Tmem231 mouse embryonic fibroblasts (MEFs) have already been described [31,40,73]. For Tmem80 immunofluorescence, cells were plated on glass coverslips, grown to confluency in DMEM+10%FBS, and starved for 48 h in OptiMEM. Cells were then fixed for 90 s at -20°C in freezer-cold methanol, incubated for 30 min at RT in block (PBS+0.1%Triton-X100+2%BSA+1% donkey serum), and incubated overnight at 4°C in block containing these primary antibodies: mouse anti-Tmem80 (Origene, TA501452, dilution 1:200), rabbit anti-detyrosinated tubulin (Millipore, AB3201, dilution 1:500), and goat anti-γ-tubulin (Santa Cruz Biotechnology, sc-7396, dilution 1:200). Coverslips were then rinsed thrice in PBS, incubated 1 h at RT with block containing DAPI and AlexaFluor-conjugated donkey secondary antibodies, rinsed twice more in PBS, mounted on slides using gelvatol, and visualised in a Leica TCS SPE confocal microscope.

## Supporting Information

S1 Fig Expression analyses and rescue of CEP-290 TZ localisation by full-length MKS-5 and coiled coil region of MKS-5. (**A**) Analysis of *cep-290* transcript abundance in wild type and *cep-290(gk415029)*. Consistent with degradation by nonsense-mediated mRNA decay (NMD) [74], we see an approximately 5-fold reduction of *cep-290* transcript level in the mutant. (**B**) Relative levels of CEP-290::GFP transcripts measured by quantitative qPCR are similar between wild-type and different TZ mutants, as indicated. (**C**) CEP-290::GFP localisation volume characteristics (in μm^3^) near or at the TZ in wild-type, *mks-5*, or MKS-5 rescue strains (*mks-5* mutant expressing full length MKS-5 or the MKS-5 coiled coil region). Results are shown as box plots, and * denotes statistically different volume. (**D-G**) Fluorescence intensity measurements of different GFP-tagged fusion proteins (TMEM-218::GFP, TMEM-231::GFP, CDKL-1A::GFP and CDKL-1C::GFP) in phasmid cell bodies of wild-type and mutant strains (as indicated) shown as box and dot plots. All transgenes are expressed at similar levels, although a few are statistically different (indicated by asterisk). *, *p* < 0.05 compared to wild-type (TMEM-218::GFP and CDKL-1A::GFP, Kruskal-Wallis test, Dunn’s method with Holm-Sidak adjustment; TMEM-231::GFP, Kruskal-Wallis test; CDKL-1C::GFP, Tukey’s test).
(EPS)

S2 Fig Rescue of the dye-filling phenotype of the *cep-290;nphp-4* double mutant strain with the wild-type CEP-290::GFP translational reporter. *cep-290;nphp-4* animals fail to uptake the lipophilic dye DiI, a phenotype rescued by low-level expression of the CEP-290::GFP construct. Shown is a transgenic animal that expresses the reporter (see arrows and inset) and can uptake the dye, while a nontransgenic sibling cannot uptake the dye (dotted circles).
(EPS)

S3 Fig Genotyping of family members with Tmem17 homozygous mutation in OFD6-affected individuals and evolutionary conservation of the mutated Tmem17 residue. (**A**) DNA Sanger sequencing results showing segregation of the homozygous C-to-A mutation (c.306C>A) causing the amino acid variation p.N102K/p.N102K. (**B**) Partial amino acid sequence alignment of Tmem17 and related proteins (Tmem80, Tmem216) in humans and divergent species showing the region encompassing the N102K mutated residue.
(EPS)

S4 Fig *TMEM138* and *TMEM231* patient mutations and *TMEM231* foetus phenotypes. (**A**) Clinical pictures of the foetus with *TMEM231* mutations at 21 wk of gestation, with asymmetric face, hypertelorism, down-slanting palpebral fissures, micrognathia, unilateral left cleft lip (a,e), hand left postaxial polydactyly (b), feet bilateral postaxial polydactyly (c,d) and normal skeletal X-rays excepting for polydactyly (f). (**B**) DNA Sanger chromatograms of the homozygous *TMEM138* c.352A>T (pM118L) mutation and the heterozygous composite *TMEM231* c656C>T / c.532C>G (pP219L / p.P178A) mutations. pest–people environment science technology SCEPTre.
(EPS)

S5 Fig Mammalian Tmem80 is a novel transition zone protein. (**A**) Phylogenetic distribution of Tmem17 superfamily proteins. While Tmem17 and a Tmem216/Tmem80-related protein are ubiquitously found across ciliated eukaryotes, only tetrapods (e.g., frogs, chickens, mammals) possess Tmem80-related orthologues. Bd, *Batrachochytrium dendrobatidis*; Cr, *Chlamydomonas reinhardtii*; Tb, *Trypanosoma brucei*, Pt, *Paramecium tetraurelia*; Ce, *Caenorhabditis elegans*; Dm, *Drosophila melanogaster*; Sp, *Strongylocentrotus purpuratus*; Od, *Oikopleura diodica*; Dr, *Danio rerio*; Gg, *Gallus gallus*; Mm, *Mus musculus*; Hs, *Homo sapiens*. (**B**) Immunofluorescence localisation of Tmem80 (red) in wild-type, homozygous mutant, or heterozygous mutant mouse embryonic fibroblasts (MEFs) for the indicated genes. Tmem80 transition zone localisation depends on several established ciliopathy-associated TZ proteins (Tctn1, Tctn2, Cc2d2a/Mks6). Antibodies against γ-tubulin (green) and detyrosinated tubulin (blue) mark the basal body and ciliary axoneme, respectively.
(EPS)

S1 Table Target sequence analysis of 101 known and candidate ciliopathy genes uncovers a homozygous mutation in *TMEM17* ^1^. (DOCX)

S2 Table Genotypes and detailed clinical features of patients with mutations in *TMEM17*, *TMEM138*, and *TMEM231*. (DOCX)

S3 Table List of *Caenorhabditis elegans* strains used in this study. (DOCX)

S4 Table Target-sequencing statistics and identification of *TMEM17*. (DOCX)

S5 Table Whole-exome statistics and identification of *TMEM138* and *TMEM231*. (DOCX)

S6 Table Primer sequences for PCR and Sanger sequencing of TMEM17, TMEM138, and TMEM231 regions with mutations. (DOCX)

## Abbreviations

BBS: Bardet-Biedl syndrome
CIZE: ciliary zone of exclusion
GFP: green fluorescent protein
IFT: intraflagellar transport
JATD: Jeune asphyxiating thoracic dystrophy
JBTS: Joubert syndrome
LCA: Leber Congenital Amaurosis
MMP: Million Mutation Project
MKS: Meckel syndrome
NGS: next generation sequencing
NMD: nonsense-mediated mRNA decay
NPHP: Nephronophthisis
OFD6: Oral-Facial-Digital syndrome type 6
SLNS: Senior Løken syndrome
TEM: transmission electron microscopy
TMEM: transmembrane
TZ: transition zone
