# Supplementary material for: MKS5 and CEP290 Dependent Assembly Pathway of the Ciliary Transition Zone
Source: PLoS Biol. 2016 Mar 16;14(3):e1002416. doi: 10.1371/journal.pbio.1002416 (PMC4794247; doi:10.1371/journal.pbio.1002416)
Supplement: S1 Table — (DOCX) [file pbio.1002416.s006.docx]

**S1 Table.** Target sequence analysis of 101 known and candidate ciliopathy genes uncovers a homozygous mutation in *TMEM17*^1^.

| *ALMS1* | *FAN1* | *NPHP4* |
| --- | --- | --- |
| *ANKS6* | *FBF1* | *PDE6D* |
| *ARL6 (BBS3)* | *GAS8* | *PIK3C2A* |
| *ATXN10* | *GLI1* | *RAB8A* |
| *B9D1 (MKSR1)* | *GLI2* | *SDCCAG8 (NPHP10)* |
| *B9D2 (MKSR2)* | *GLI3* | *SLC41A1* |
| *BBIP10* | *GLIS2 (NPHP7)* | *SSNA1* |
| *BBS1* | *GLIS3* | *SUFU* |
| *BBS10 (FLJ23560; C12orf58)* | *HYLS1* | *TCTN1* |
| *BBS12 (FLJ35630; C4orf24)* | *IFT122* | *TCTN3* |
| *BBS2* | *IFT140* | *TEKT1* |
| *BBS4* | *IFT172* | *TEKT2* |
| *BBS5* | *IFT43* | *TEKT3* |
| *BBS6 (MKKS)* | *IFT80* | *TEKT4* |
| *BBS7* | *INPP5B* | *TEKT5* |
| *BBS9 (PTHB1)* | *IQCB1 (NPHP5)* | *TMEM138* |
| *C12orf38 (TCTN2)* | *JADE1* | ***TMEM17*** *–* p. N102K mutation |
| *C5ORF42* | *JBTS1 (INPP5E)* | *TMEM231* |
| *CCDC28B* | *JBTS10 (OFD1; CXORF5)* | *TMEM237 (ALS2CR4)* |
| *CDC42* | *JBTS2 (TMEM216; MKS2)* | *TMEM80* |
| *CEP162* | *JBTS3 (AHI1)* | *TMEM218* |
| *CEP164* | *JBTS6 (MKS3; TMEM67)* | *TRAM1* |
| *CEP290 (BBS14; JBTS5; MKS4; NPHP6)* | *JBTS7 (MKS5,NPHP8; RPGRIP1L)* | *TRIM32 (BBS11)* |
| *CEP41 (TSGA14)* | *JBTS8 (ARL13B)* | *TTC21B (JBTS11)* |
| *CP110* | *JBTS9 (MKS6; KIAA1345; CC2D2A)* | *TTC8 (BBS8)* |
| *CROCC* | *KIF14* | *WDPCP (BBS15; C2ORF86)* |
| *CSPP1* | *KIF7* | *WDR19 (IFT144)* |
| *DYNC2H1* | *MAPKAP1 (SIN1)* | *WDR34* |
| *DYX1C1* | *MKS1 (BBS13)* | *WDR35* |
| *DZIP1* | *MRE11A* | *WDR60* |
| *EFHC1* | *NEK1* | *ZNF423* |
| *EVC* | *NEK8 (NPHP9)* |  |
| *EVC2* | *NPHP1 (JBTS4)* |  |
| *EXOC5 (SEC10)* | *NPHP2 (INVS)* |  |
| *EXOC8* | *NPHP3 (MKS7)* |  |

^1^Cells highlighted in green represent genes encoding established ciliary transition zone (TZ) proteins. *TMEM17* is the only gene found to have a deleterious homozygous mutation affecting the coding region (p.N102K).
