# Supplementary material for: MKS5 and CEP290 Dependent Assembly Pathway of the Ciliary Transition Zone
Source: PLoS Biol. 2016 Mar 16;14(3):e1002416. doi: 10.1371/journal.pbio.1002416 (PMC4794247; doi:10.1371/journal.pbio.1002416)
Supplement: S2 Table — (DOCX) [file pbio.1002416.s007.docx]

**S2 Table.** Genotypes and detailed clinical features of patients with mutations in *TMEM17*, *TMEM138* and *TMEM231*.

| **Genotype** | | | | |
| --- | --- | --- | --- | --- |
| Gene | | *TMEM17* | *TMEM138* | *TMEM231* |
| Transcript ID | | NM_198276.2 | NM_016464.4 | NM_001077416.2 |
| DNA Mutation | | c.306C>A  c.306C>A | c.352A>T  c.352A>T | c.656C>T  c.532C>G |
| Protein Change | | p.Asn102Lys  p.Asn102Lys | p.Met118Leu  p.Met118Leu | p.Pro219Leu  p.Pro178Ala |
| OMIM | | Not assigned yet | JS [MIM 614465] | JS [MIM 614970]  MS [MIM 615397]  OFDS |
| **Phenotype** | | | | |
| Age at last follow-up | | 36 / 20 years | 9 years | 21 wg |
| Sex | | M / M | F | M |
| Consanguinity | | - | + | - |
| Oral | Cleft palate | -/- | + | + |
|  | Pierre-Robin sequence | -/- | + | - |
|  | Lingual hamartomas | +/+ | + | + |
|  | Bifid tongue | -/- | + | - |
|  | Abnormal frenulae | +/+ | + | - |
|  | Tooth abnormalities | -/- | + | NA |
|  | Micro/retrognathia | -/- | + | + |
| Facial | Facial dysmorphism | - /- | + | + |
|  | Hypertelorism | +/+ | + | + |
|  | Hypoplasic nose/alae | -/- | + | - |
|  | Median cleft | -/- | + | - |
|  | Cleft lip | + (notched)/- | - | + |
| Digital | Hands | -/Bilat. postaxial polydactyly | Bilat. brachydactyly  L III-IV syndactyly, III brachymesophalangy, V clinodactyly  R IV clinodactyly | Bilat. postaxial polydactyly |
|  | Feet | L postaxial polydactyly / Bilat. postaxial polydactyly | L II-III syndactyly and III-IV-V overlapping | Bilat. postaxial polydactyly |
|  | Asymmetric signs | -/- | + | - |
| Brain | Corpus callosum agenesis | -/- | + | - |
|  | Cerebellar vermis agenesis | +/+ | + | - |
|  | Cerebellar hypoplasia | +/+ | - | + |
|  | Molar tooth sign | +/+ | - | NA |
|  | Dandy-Walker Malformation | -/- | - | + |
|  | Nodular grey matter heterotopia | -/- | + | - |
| Other | Short stature | -/- | - | - |
|  | 12^th^ rib hypoplasia | -/- | - | + |
|  | Polycystic kidney disease | -/- | - | - |
|  | Miliae | -/- | + | - |
|  | Psychomotor delay | +/+ | + | NA |

Bilat: Bilateral; JS: Joubert syndrome; MS: Meckel syndrome; OFDS: Oral-facial-digital syndrome; NA: Not Available; wg: weeks of gestation
