# Supplementary material for: MKS5 and CEP290 Dependent Assembly Pathway of the Ciliary Transition Zone
Source: PLoS Biol. 2016 Mar 16;14(3):e1002416. doi: 10.1371/journal.pbio.1002416 (PMC4794247; doi:10.1371/journal.pbio.1002416)
Supplement: S3 Table — (DOCX) [file pbio.1002416.s008.docx]

**S3 Table**. List of *C. elegans* strains used in this study.

| **Strain** | **Genotype** |
| --- | --- |
| N2 | *Bristol wild-type* |
| MX067 | *Ex[tmem-138::gfp; Posm-5::xbx-1::tdtomato; rol-6(su1006)]* |
| MX735 | *nphp-4(tm925)* |
| MX771 | *nphp-1(ok500)* |
| MX1065 | *Ex[tmem-17::gfp; Posm-5::xbx-1::tdtomato; rol-6(su1006)]* |
| MX1251 | *mks-2(nx111)* |
| MX1388 | *Ex[arl-13::gfp; Posm-5::xbx-1::tdtomato; rol-6(su1006)]* |
| MX1415 | *Ex[tmem-231:gfp; Posm-5::xbx-1::tdTomato; rol-6(su1006)]* |
| MX1420 | *Ex[Pbbs-8::tram-1::tdtomato; Pbbs-8::mks-2::gfp; rol-6(su1006)]* |
| MX1534 | *tmem-138(tm5624)* |
| MX1915 | *mks-5(tm3100); Ex[Pbbs-8::cdkl-1C::gfp; Posm-5::xbx-1::tdtomato; rol-6(su1006)]* |
| MX1919 | *mks-2(nx111); Ex[Pbbs-8::cdkl-1C::gfp; Posm-5::xbx-1::tdtomato; rol-6(su1006)]* |
| MX1920 | *cdkl-1(tm4182); mks-2(nx111)* |
| MX1929 | *Ex[cdkl-1A::gfp; Posm-5::xbx-1::tdtomato; rol-6(su1006)]* |
| MX1932 | *Ex[rpi-2::gfp; mksr-1::tdtomato; rol-6(su1006)]* |
| MX1945 | *cep-290(gk415029); Ex[Pbbs-8::tram-1::tdtomato; Pbbs-8::mks-2::gfp; rol-6(su1006)]* |
| MX1947 | *Ex[tmem-218::gfp; Posm-5::xbx-1::tdtomato; rol-6(su1006)]* |
| MX1957 | *cep-290(gk415029); Ex[cdkl-1A::gfp; Posm-5::xbx-1::tdtomato; rol-6(su1006)]* |
| MX1958 | *cep-290(gk415029); Ex[Posm-5::mks-5::tdtomato; Posm-5::dyf-11::gfp; rol-6(su1006)]* |
| MX1959 | *cep-290(gk415029)* |
| MX1961 | *mks-5(tm3100); Ex[tmem-218::gfp; Posm-5::xbx-1::tdtomato; rol-6(su1006)]* |
| MX1962 | *nphp-4(tm925); Ex[tmem-218::gfp; Posm-5::xbx-1::tdtomato; rol-6(su1006)]* |
| MX1963 | *mks-2(nx111); Ex[tmem-218::gfp; Posm-5::xbx-1::tdTomato; rol-6(su1006)]* |
| MX1964 | *cep-290(gk415029); Ex[rpi-2::gfp; mksr-1::tdtomato; rol-6(su1006)]* |
| MX1965 | *cep-290(gk415029); Ex[arl-13::gfp; Posm-5::xbx-1::tdtomato; rol-6(su1006)]* |
| MX1966 | *cep-290(gk415029); Ex[nphp-1::cfp; che-13::yfp; rol-6(su1006)]* |
| MX1967 | *cep-290(gk415029); Ex[nphp-4::yfp; che-13::cfp; rol-6(su1006)]* |
| MX1980 | *Ex[Pbbs-8::cep-290cDNA::gfp+ Posm-5::xbx-1::tdTomato + rol-6(su1006)]* |
| MX1989 | *mks-2(nx111); Ex[Pbbs-8::cep-290cDNA::gfp; Posm-5::xbx-1::tdtomato; rol-6(su1006)]* |
| MX1990 | *mks-5(tm3100); Ex[Pbbs-8::cep-290cDNA::gfp; Posm-5::xbx-1::tdtomato; rol-6(su1006)]* |
| MX1991 | *nphp-4(tm925); Ex[Pbbs-8::cep-290cDNA::gfp; Posm-5::xbx-1::tdtomato; rol-6(su1006)]* |
| MX2029 | *Ex[Pbbs-8::cdkl-1C::gfp; Posm-5::xbx-1::tdtomato; rol-6(su1006)]* |
| MX2101 | *cdkl-1(tm4182); nphp-1(ok500)* |
| MX2102 | *cdkl-1(tm4182)* |
| MX2168 | *cdkl-1(tm4182); tmem-138(tm5624)* |
| MX2172 | *tmem-231(tm5963); Ex[Pbbs-8::cdkl-1C::gfp; Posm-5::xbx-1::tdtomato; rol-6(su1006)]* |
| MX2261 | *cep-290(gk415029); Ex[tmem-138::gfp; Posm-5::xbx-1::tdtomato; rol-6(su1006)]* |
| MX2262 | *tmem-218(nx114)* |
| MX2263 | *tmem-218(nx114); Ex[Pbbs-8::tram-1::tdtomato; Pbbs-8::mks-2::gfp; rol-6(su1006)]* |
| MX2264 | *tmem-218(nx114); Ex[nphp-4::yfp; che-13::cfp; rol-6(su1006)]* |
| MX2265 | *tmem-218(nx114); Ex[Pbbs-8::cep-290cDNA::gfp; Posm-5::xbx-1::tdtomato; rol-6(su1006)]* |
| MX2266 | *tmem-218(nx114); Ex[Posm-5::mks-5::tdtomato; Posm-5::dyf-11::gfp; rol-6(su1006)]* |
| MX2267 | *cep-290(gk415029); Ex[tmem-231::gfp; Posm-5::xbx-1::tdtomato; rol-6(su1006)]* |
| MX2268 | *cep-290(gk415029); Ex[tmem-17::gfp; Posm-5::xbx-1::tdtomato; rol-6(su1006)]* |
| MX2269 | *cep-290(gk415029); Ex[tmem-218::gfp; Posm-5::xbx-1::tdtomato; rol-6(su1006)]* |
| MX2297 | *tmem-218(nx114); nphp-4(tm925)* |
| MX2298 | *cep-290(gk415029); nphp-4(tm925)* |
| MX2300 | *nphp-4(tm925); Ex[cdkl-1A::gfp; Posm-5::xbx-1::tdtomato; rol-6(su1006)]* |
| MX2330 | *mks-2(nx111); cep-290(gk415029)* |
| MX2331 | *mks-2(nx111); tmem-218(nx114)* |
| MX2343 | *cep-290(gk415029); nphp-4(tm925); Ex[Pbbs-8::cep-290cDNA::gfp; rol-6(su1006)]* |
| MX2367 | *cep-290(gk415029); cdkl-1(tm4182)* |
| MX2368 | *cep-290(gk415029); tmem-138(tm5624)* |
| MX2388 | *mks-5(tm3100); Ex[Pbbs-8::cep-290cDNA::gfp, mks-5::tdTomato]* |
| MX2389 | *mks-5(tm3100); Ex[Pbbs-8::cep-290cDNA::gfp, mks-5(Δaa565-1306)::tdTomato]* |
| YH224 | *Ex[nphp-4::yfp; che-13::cfp; rol-6(su1006)]* |
| YH237 | *Ex[nphp-1::cfp; che-13::yfp; rol-6(su1006)]* |
| YH930 | *Ex[Posm-5::mks-5::tdtomato; Posm-5::dyf-11::gfp; rol-6(su1006)]* |
