# Supplementary material for: MKS5 and CEP290 Dependent Assembly Pathway of the Ciliary Transition Zone
Source: PLoS Biol. 2016 Mar 16;14(3):e1002416. doi: 10.1371/journal.pbio.1002416 (PMC4794247; doi:10.1371/journal.pbio.1002416)
Supplement: S4 Table — (DOCX) [file pbio.1002416.s009.docx]

**S4 Table**. Target-sequencing statistics and identification of *TMEM17*.

| Ciliary-related genes included in the panel | 120 |
| --- | --- |
| Number of reads | 1824596 |
| Percent of mapped reads (%) | 96.2 |
| Percent of duplicate reads (%) | 4,9 |
| Rare variants (exonic + splicing regions) | 6 |
| Homozygous variants | 1 |
| Causal gene | *TMEM17* |
