# Supplementary material for: MKS5 and CEP290 Dependent Assembly Pathway of the Ciliary Transition Zone
Source: PLoS Biol. 2016 Mar 16;14(3):e1002416. doi: 10.1371/journal.pbio.1002416 (PMC4794247; doi:10.1371/journal.pbio.1002416)
Supplement: S5 Table — (DOCX) [file pbio.1002416.s010.docx]

**S5 Table**. Whole-exome statistics and identification of *TMEM138* and *TMEM231*.

|  | **case 1** | **case 2** |
| --- | --- | --- |
| Number of reads | 52993416 | 49790276 |
| Percent of mapped reads (%) | 99 | 99,6 |
| Percent of pairing (%) | 98,9 | 96,6 |
| Percent of duplicate reads (%) | 0,29 | 0,03 |
| Rare variants | 278 | 287 |
| Truncating variants | 47 | 17 |
| OMIM genes | 67 | 72 |
| Ciliary genes | 103 | 59 |
| Truncating Ciliary genes | 14 | 0 |
| Recessive Ciliary genes | 13 | 1 |
| Homozygous Ciliary genes | 1 | 0 |
| Causal gene | *TMEM138* | *TMEM231* |
