# Supplementary material for: MKS5 and CEP290 Dependent Assembly Pathway of the Ciliary Transition Zone
Source: PLoS Biol. 2016 Mar 16;14(3):e1002416. doi: 10.1371/journal.pbio.1002416 (PMC4794247; doi:10.1371/journal.pbio.1002416)
Supplement: S6 Table — (DOCX) [file pbio.1002416.s011.docx]

**S6 Table**. Primer sequences for PCR and Sanger sequencing of TMEM17, TMEM138 and TMEM231 regions with mutations.

| **Gene** | **Exon** | **Forward Primer** | **Reverse Primer** |
| --- | --- | --- | --- |
| *TMEM17* | 3 | CCCTGACGTTGTTAGAGTGTC | CCCCATTTTCTTATTCAGGATCC |
| *TMEM138* | 4 | AAAGTCTTTATCTGACAGGAGCTGA | TCCTCATTTCCCATAGTCTTCTAGC |
| *TMEM231* | 3 | GGGATTTAACTTAGGCCAATGGAAT | GCAATTTAGTTCTTCGACAAACACA |
| *TMEM231* | 2 | CCATTTATTTTGGCCAGTGAACATT | TAGAGTGCGGGTCTTCTAGAAATC |
